# Blockade of AMPK-Mediated cAMP–PKA–CREB/ATF1 Signaling Synergizes with Aspirin to Inhibit Hepatocellular Carcinoma

**DOI:** 10.3390/cancers13071738

**Published:** 2021-04-06

**Authors:** Hongying Zhang, Songpeng Yang, Jiao Wang, Yangfu Jiang

**Affiliations:** 1 Laboratory of Oncogene, State Key Laboratory of Biotherapy, West China Hospital, Sichuan University, Chengdu 610041, China; zhanghongying92@163.com (H.Z.); iamysp@126.com (S.Y.); 2 School of Basic Medicine, Chengdu University of Traditional Chinese Medicine, Chengdu 610075, China; wangjiao@cdutcm.edu.cn

**Keywords:** acetylsalicylic acid, aspirin, AMPK, CREB, drug–drug synergism, liver cancer

## Abstract

**Simple Summary:**

Epidemiological and experimental studies have demonstrated that aspirin (acetylsalicylic acid) may prevent the incidence of some types of human cancer, including colorectal cancer and hepatocellular carcinoma (HCC). In addition, preclinical studies indicate that aspirin in combination with other treatments may achieve a more significant anti-cancer effect for established tumors. This study aims to improve the anti-cancer effect of aspirin by targeting signaling pathways related to aspirin and its targets. We find that aspirin may induce cAMP–PKA–CREB/ATF1 signaling in HCC via AMPK and its downstream target carbamoyl-phosphate synthase 1 (CPS1). Blockade of PKA–CREB/ATF1 signaling by the natural agent berbamine could sensitize HCC to aspirin. This research indicates that the combination of two inexpensive drugs, aspirin and berbamine, holds promise in preventing and treating HCC.

**Abstract:**

Aspirin can prevent or inhibit inflammation-related cancers, such as colorectal cancer and hepatocellular carcinoma (HCC). However, the effectiveness of chemotherapy may be compromised by activating oncogenic pathways in cancer cells. Elucidation of such chemoresistance mechanisms is crucial to developing novel strategies to maximize the anti-cancer effects of aspirin. Here, we report that aspirin markedly induces CREB/ATF1 phosphorylation in HCC cells, which compromises aspirin’s anti-HCC effect. Inhibition of AMP-activated protein kinase (AMPK) abrogates the induction of CREB/ATF1 phosphorylation by aspirin. Mechanistically, activation of AMPK by aspirin results in decreased expression of the urea cycle enzyme carbamoyl-phosphate synthase 1 (CPS1) in HCC cells and xenografts. Treatment with aspirin or CPS1 knockdown stimulates soluble adenylyl cyclase expression, thereby increasing cyclic AMP (cAMP) synthesis and stimulating PKA–CREB/ATF1 signaling. Importantly, abrogation of aspirin-induced CREB/ATF1 phosphorylation could sensitize HCC to aspirin. The bis-benzylisoquinoline alkaloid berbamine suppresses the expression of cancerous inhibitor of protein phosphatase 2A (CIP2A), leading to protein phosphatase 2A-mediated downregulation of CREB/ATF1 phosphorylation. The combination of berbamine and aspirin significantly inhibits HCC in vitro and in vivo. These data demonstrate that the regulation of cAMP-PKA-CREB/ATF1 signaling represents a noncanonical function of CPS1. Targeting the PKA–CREB/ATF1 axis may be a strategy to improve the therapeutic effects of aspirin on HCC.

## 1. Introduction

Epidemiological and experimental studies have demonstrated that the nonsteroidal anti-inflammatory agent aspirin (acetylsalicylic acid) may prevent the incidence of human cancer, such as colorectal cancer and hepatocellular carcinoma (HCC) [1,2,3]. Aspirin use not only reduces the risk for liver fibrosis, an established premalignant disease [4], but also reduces the risk for HCC in hepatitis B/C virus carriers [5,6]. In addition, preclinical studies indicate that aspirin, especially in combination with sorafenib, can suppress HCC growth and metastasis [7,8,9,10]. The mechanisms underpinning the anti-cancer effects of aspirin are complex. Inhibition of platelet activation may contribute, in part, to the chemopreventive and antimetastatic properties of aspirin [11]. While cyclooxygenases (COX) and NFĸB are well-known targets of aspirin for its platelet-suppressive and cancer-preventive effects, aspirin also regulates the activity of AMP-activated protein kinase (AMPK) and PFKFB3 [7,9,12,13,14]. Moreover, the acetyl group of aspirin may be transferred to proteins, such as COX1/2 and histone, leading to protein acetylation and changed activity [15,16]. In contrast, aspirin may inhibit the acetylation of some proteins by suppressing the acetyltransferase EP300 [17].

As one of the targets of aspirin, AMPK is an evolutionarily conserved energy sensor and a signal transducer that regulates energy homeostasis, autophagy and redox balance in diverse organisms [18]. Hence, AMPK is an attractive therapeutic target for metabolic diseases and cancer [19]. However, accumulating evidence indicates that AMPK has multiple substrates in diverse processes [20] and plays tumor-suppressive or tumor-promoting roles in a context-dependent manner [21]. AMPK can inhibit mTOR and protein synthesis by phosphorylating tuberous sclerosis complex 2 (TSC2) and raptor, which may contribute to aspirin’s anti-cancer effects [13,14,22,23]. In addition, AMPK promotes CDKN1A/B and p53 phosphorylation, thereby inducing cell cycle arrest [24]. On the other hand, AMPK may inhibit stresses-induced cell death. For example, AMPK can relieve oxidative stress by inhibiting fatty acid synthesis [25]. The growth of Kras-mutated lung cancer is promoted by AMPK through activating the TFE3 transcription factor, thereby stimulating lysosome biogenesis [26]. Given that AMPK has both tumor-suppressive and tumor-promoting effects, blockade of the unwanted tumor-promoting effects of AMPK may potentiate the anti-cancer effects of aspirin.

A previous study demonstrates that AMPK downregulates carbamoyl phosphate synthase 1 (CPS1) in Kras-mutated non-small-cell lung cancer (NSCLC) cells [27]. CPS1, ornithine transcarbamylase, argininosuccinate synthase, argininosuccinate lyase and arginase drive the urea cycle, a major metabolic pathway to prevent ammonia toxicity [28]. Except for the disposal of excess nitrogen, multiple metabolic intermediates are produced in the urea cycle to enhance polyamines and pyrimidine synthesis and stimulate cancer cell proliferation. Although ammonia is a highly toxic metabolic product [29], it is an important nitrogen source of tumor cells that are recycled and incorporated into glutamate to support the synthesis of lipids, amino acids, nucleotides and antioxidants [30]. CPS1 is only expressed in the liver but not other healthy tissues. However, CPS1 is overexpressed in a subset of non-small cell lung cancer (NSCLC), colon cancer, glioblastoma and cholangiocarcinoma, in which it is associated with poor prognosis [27,31]. In contrast, CPS1 expression is downregulated in HCC [32]. The detrimental effects of CPS1 loss on HCC remain to be characterized.

Thus far, it is unclear whether AMPK regulates CPS1 in HCC. In addition, the effect of aspirin on CPS1 remains elusive. Here, we report that aspirin inhibits CPS1 expression in HCC through activating AMPK. Importantly, downregulation of CPS1 leads to an increase in the levels of cyclic AMP (cAMP) and activation of protein kinase A (PKA)-cAMP response element-binding protein (CREB)/ATF1 axis, which compromises the anti-cancer effects of aspirin. Previous studies have demonstrated that cAMP–PKA–CREB signaling has important roles in various human cancers [33]. Moreover, PKA activation is regulated by other oncogenic protein kinases, such as insulin-like growth factor receptors [34]. Our current study demonstrates that the regulation of cAMP/PKA/CREB signaling represents a noncanonical function of CPS1. In addition, abrogation of aspirin-induced CREB/ATF1 phosphorylation can sensitize HCC to aspirin. Berbamine, a kind of bis-benzylisoquinoline alkaloid [35], can suppress CREB/ATF1 phosphorylation and synergize with aspirin to inhibit HCC.

## 2. Materials and Methods

The effects of aspirin on AMPK and CREB/ATF1 phosphorylation, CPS1 and sAC expression in HCC cells were detected by Western blotting. The effects of aspirin on CREB activity were detected by CREB luciferase reporter assay. In addition, we took advantage of RNA interference to detect the involvement of AMPK in aspirin-induced CREB/ATF1 phosphorylation, c-Jun transcription, CPS1 and sAC expression, and increase in intracellular cAMP levels. Quantitative reverse transcription-PCR was used to detect aspirin’s effects on c-Jun, c-Fos, CPS1 and sAC transcription. In addition, the effect of aspirin or CPS1 knockdown on PKA activity was detected by a non-radioactive cAMP-dependent protein kinase assay. The effect of aspirin or CPS1 knockdown on intracellular cAMP concentration was detected by ELISA. CCK-8, EdU and colony formation assays were used to detect the effects of aspirin and berbamine on HCC cell viability and proliferation. The effect of aspirin and berbamine on HCC growth in vivo was determined in a xenograft mouse model. Finally, the effects of aspirin and berbamine on tumor cell proliferation, CPS1 expression and CREB/ATF1 phosphorylation were detected by immunohistochemical analysis of xenograft tumors.

### 2.1. Cell Culture

HepG2 (RRID:CVCL_0027) and Hep3B (RRID:CVCL_0326) cell lines were obtained from Cell Lines Bank, Chinese Academy of Science (Shanghai, China). The cells were cultured in Dulbecco’s modified Eagle’s medium (DMEM) containing 10% FBS, 100 mg/mL penicillin G, and 50 g/mL streptomycin at 37 °C in a humidified atmosphere containing 5% CO_2._ Experiments were performed within 20 passages after cell thawing.

### 2.2. Reagents and Antibodies

Details are presented in Tables S1 and S2.

### 2.3. RNA Interference

All siRNAs were purchased from GenePharma (Shanghai, China). Proliferating cells were incubated with 50 nM siRNA in serum-free DMEM containing lipofectamine 2000. 4–6 h later, cells were incubated in complete DMEM for 48 h, followed by further experiments. The siRNA sequences are shown in Table S3.

### 2.4. Quantitative Real-Time PCR Analysis

A cell total RNA isolation kit (Foregene, Chengdu, China) was utilized to extract total RNAs from cultured cells. Subsequently, RNA was reversely transcribed into cDNAs using oligo dT primers and the HiScript Q RT SuperMix (Vazyme, Nanjing, China). The quantitative real-time PCR assay was carried out using the SYBR master mix (Vazyme). The mRNA levels of GAPDH were detected as an internal control. All primer sequences are shown in Table S4.

### 2.5. Western Blotting

Total protein was extracted using RIPA buffer supplemented with protease inhibitors and a phosphatase inhibitor cocktail. About 30 μg of proteins were subjected to SDS–PAGE, followed by transferring to PVDF membrane and incubating with primary or secondary antibodies as previously described [9]. Detection was performed with BeyoECL Plus (Beyotime, Shanghai, China). Images were taken by Fusion Fx (Vilber Lourmat, France) imaging system. The blots were quantified by Image J software. File S1 shows the original Western blots used for this manuscript.

### 2.6. CREB Luciferase Reporter Assay

Cells were seeded in 24-well plates and then transfected with the CREB luciferase reporter plasmid that contains multiple CREB-binding sites, while other potential transcription factors-binding sites were mutated (Genomeditech, Shanghai, China). As an internal control, the renilla luciferase plasmid (pRL-TK) was co-transfected. The cells were treated with or without 5 mM aspirin for 24 h. The luciferase activities were measured using a dual-luciferase reporter assay kit (Vazyme, Nanjing, China). The relative CREB reporter activities were normalized by the renilla luciferase values.

### 2.7. Intracellular cAMP Measurements

HepG2 cells were treated with vehicle or 5 mM aspirin for 1 h prior to measuring cAMP. For CPS1 knockdown, the cells were treated with siControl or siCPS1 for 48 h prior to measuring cAMP. For AMPKα1 knockdown, cells were treated with siControl or siAMPKα1 for 48 h, followed by treatment with or without 5 mM aspirin for 1 h prior to measuring cAMP. After treatment, intracellular cAMP concentration was detected with the cyclic AMP ELISA Kit (Cayman Chemical, Ann Arbor, MI, USA) according to the manufacturer’s protocol. Briefly, cells were washed with PBS and incubated with 0.1 M HCl at room temperature. Twenty minutes later, the cells were scraped off the plate and centrifuged at 1000× *g* to collect the supernatant. The supernatant was diluted with ELISA buffer for neutralization. Fifty microliters of the supernatant were used for measurement. The plate was incubated at 4 °C for 18 h, followed by developing with Ellman’s Reagent in the dark for 90 min. Absorbance was detected by a microplate reader at a wavelength of 420 nm. Protein concentrations of cell lysates were measured with BCA Protein Assay. The cAMP levels were normalized to protein concentrations.

### 2.8. PKA Activity Assay

HepG2 cells were treated with or without aspirin for 48 h prior to measuring PKA activity. For CPS1 knockdown, the cells were transfected with siControl or siCPS1 for 48 h. After treatment, the cells were suspended in a cold PKA extraction buffer and homogenized. The lysate was centrifuged at 14,000× *g* for 5 min at 4 °C, and the supernatant was saved. PKA activity was detected by the PepTagR non-radioactive cAMP-dependent protein kinase assay (Promega, Madison, WI, USA) according to the manufacturer’s protocol. The phosphorylated and non-phosphorylated dye-labeled kemptide were separated in a 0.8% agarose gel. The gel was photographed on a UV-illuminator. In addition, the phosphorylated band in the gel was cut and dissolved in Gel Solubilization Solution and glacial acetic acid, followed by detecting the fluorescence values at the ex/em wavelength of 530/590 nm.

### 2.9. Cell Growth Assay

Cells were seeded in a 96-well plate. After treating with aspirin and/or berbamine, the cells were washed with PBS, followed by adding CCK8 reagent (Dojindo Laboratory, Kumamoto, Japan) into the cultures. Two hours later, the absorbance was measured by a microplate reader at 450 nm wavelength. The IC_50_ values were calculated using the GraphPad Prism software. The synergism of aspirin and berbamine in inhibiting HCC cell growth was determined. The combination index (CI) values were calculated using the CalcuSyn software. Synergism was indicated by a CI value less than 0.9. The less CI value indicated a stronger synergism.

### 2.10. Colony Formation Assay

HepG2 cells were seeded in 6-well plates at 3000 cells per well. The cells were treated with or without 2.5 mM aspirin and 5 μM berbamine. 48 h later, the cells were incubated in the absence of drugs and allowed to form colonies for 7 days. The colonies were stained with 2% crystal violet and then counted. The colony number was analyzed by Image J.

### 2.11. EdU Assay

Cells seeded in 96-well plates were treated with 5 mM aspirin with or without 35 μM berbamine for 48 h. After treatment, EdU staining was performed using a Cell-Light^TM^ EdU Apollo567 in vitro kit (RiboBio, Guangzhou, China). The percentage of proliferating cells was quantified by analyzing the images in random fields.

### 2.12. Xenograft Tumor Growth

HepG2 cells (4 × 10^6^ in 125 μL PBS) were subcutaneously injected into 5-week-old BALB/c nude mice. About one week later, the mice with tumors were randomly divided into 4 groups, with 8 mice per group. The mice were orally administrated with aspirin (100 mg/kg/day), berbamine (50 mg/kg/day) or both as previously described [7,8,9,13,33]. The control group was treated with a vehicle. Tumor size was measured every 3 days. Tumor volume (v) was calculated using the formula: v = (length × width^2^)/2. Twenty-six days after inoculation of cancer cells, all mice were sacrificed. The tumors were collected and stored for subsequent analysis. All procedures adhered to the guidelines approved by the Animal Ethics Committee of the West China Hospital of Sichuan University (approval No. 2020227A).

### 2.13. Immunohistochemistry

Immunohistochemistry (IHC) of tumor tissue sections was performed as previously described [9]. Briefly, histologic sections were mounted on slides and deparaffinized, followed by rehydration. The slides were incubated with anti-CPS1, anti-PCNA or anti-p-CREB/ATF1 antibody overnight at 4 °C, and then incubated with secondary antibody at room temperature. The slides were further incubated with streptavidin/horseradish peroxidase for 15 min, stained with diaminobenzidine chromogen, and counterstained with hematoxylin.

### 2.14. Statistical Analysis

One-way analysis of variance with post hoc tests was used in statistical analysis of mRNA expression, cAMP levels, PKA activity, cell proliferation rate, and colony number. CREB luciferase reporter activity, tumor volume and weight were statistically analyzed by unpaired Student’s t-tests (two-tailed). All statistical analyses were conducted using GraphPad Prism, version 7.0. Differences were considered statistically significant if *p* < 0.05.

## 3. Results

### 3.1. Aspirin Induces CREB/ATF1 Phosphorylation and Activation in HCC Cells through AMPK

**Figure 1:**
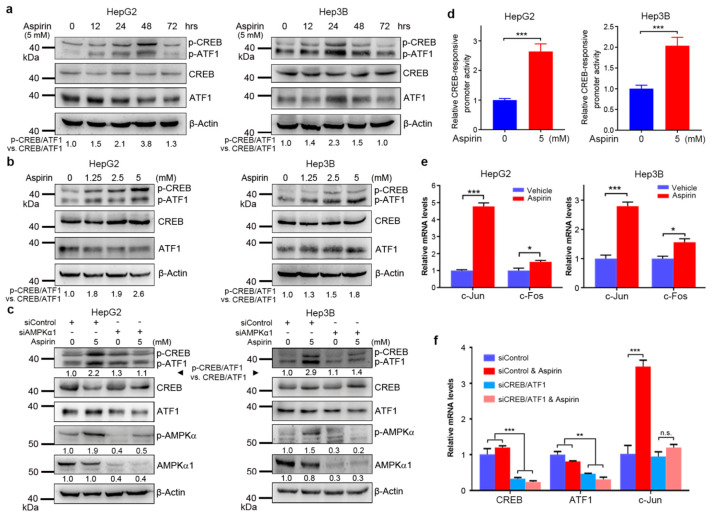
Aspirin induces (PKA)-cAMP response element-binding protein (CREB /ATF1) activation through AMP-activated protein kinase (AMPK). (**a**) HepG2 and Hep3B cells were treated with aspirin for indicated periods, followed by western blot analysis with indicated antibodies. The relative levels of p-CREB/ATF1 normalized to total CREB/ATF1 were shown. (**b**) HepG2 and Hep3B cells were treated with indicated doses of aspirin for 32 h, followed by western blot analysis with indicated antibodies. The relative levels of p-CREB/ATF1 normalized to total CREB/ATF1 were shown. (**c**) HepG2 and Hep3B cells were transfected with siControl or siAMPKα1 and treated with or without aspirin for 32 h, followed by western blot analysis with indicated antibodies. The relative levels of p-CREB/ATF1 normalized to total CREB/ATF1, and the relative levels of p-AMPK and AMPKα1 normalized to β-actin were shown. (**d**) HepG2 and Hep3B cells were transfected with the CREB luciferase reporter plasmid and the renilla luciferase plasmid, followed by treatment with or without aspirin for 24 h. The luciferase activities were measured using a dual-luciferase reporter assay kit. The CREB-responsive promoter activity was normalized by the renilla luciferase values. The relative CREB-responsive promoter activity was plotted. Values represent means ± SD (*n* = 4). ***, *p* < 0.001. (**e**) HepG2 and Hep3B cells were treated with or without 5 mM aspirin for 48 h, followed by real-time RT–PCR analysis of c-Jun and c-Fos transcription. The relative mRNA levels were plotted. Values represent means ± SD (*n* = 3). *, *p* < 0.05, ***, *p* < 0.001. (**f**) HepG2 cells were transfected with siControl or siCREB/ATF1 and treated with or without 5 mM aspirin, followed by real-time RT–PCR analysis of indicated genes. The relative mRNA levels were plotted. Values represent means ± SD (*n* = 3). **, *p* < 0.01. ***, *p* < 0.001. *n.s.*, not significant.

The transcription factors CREB and its family member ATF1 may promote tumorigenesis and chemoresistance and can be activated by PKA [33]. PKA activity can be promoted by both COX-prostaglandin E2 (PGE2)-EP2 receptor signaling and AMPK [36,37]. Since aspirin is a COX inhibitor and an AMPK activator [1,9,12,13], it needs to determine how aspirin may eventually regulate CREB/ATF1 in HCC cells. To this end, HepG2 cells were treated with or without aspirin, followed by Western blot analysis of CREB/ATF1 phosphorylation. Aspirin induced the phosphorylation of CREB and ATF1 in a time- and dose-dependent manner (Figure 1a,b). Similar effects were detected in Hep3B cells (Figure 1a,b). Treatment of HepG2 cells with the AMPK agonist AICAR also resulted in CREB/ATF1 phosphorylation (Figure S1). Moreover, AMPKα1 knockdown abrogated the induction of CREB/ATF1 phosphorylation by aspirin in both HepG2 and Hep3B cells (Figure 1c). Together, these data demonstrate that aspirin induces CREB/ATF1 phosphorylation through AMPK.

Next, we detected the effects of aspirin on CREB activity using a CREB luciferase reporter that harbors multiple CREB-binding sites. Treatment of HepG2 and Hep3B cells with aspirin significantly increased the CREB-responsive promoter activity (Figure 1d). In addition, we detected the effects of aspirin on CREB/ATF1 targets. Treatment of HepG2 and Hep3B cells with aspirin led to a drastic increase in c-Jun transcription and a moderate increase in c-Fos transcription (Figure 1e). Knockdown of CREB and ATF1 abrogated the induction of c-Jun transcription by aspirin (Figure 1f). Moreover, AMPKα1 knockdown abrogated the induction of c-Jun transcription by aspirin (Figure S2). Together, these data indicate that aspirin upregulates the transcriptional activity of CREB/ATF1.

### 3.2. AMPK-Mediated Increase in cAMP Levels and PKA Activity Contributes to the Induction of CREB/ATF1 Phosphorylation by Aspirin

**Figure 2:**
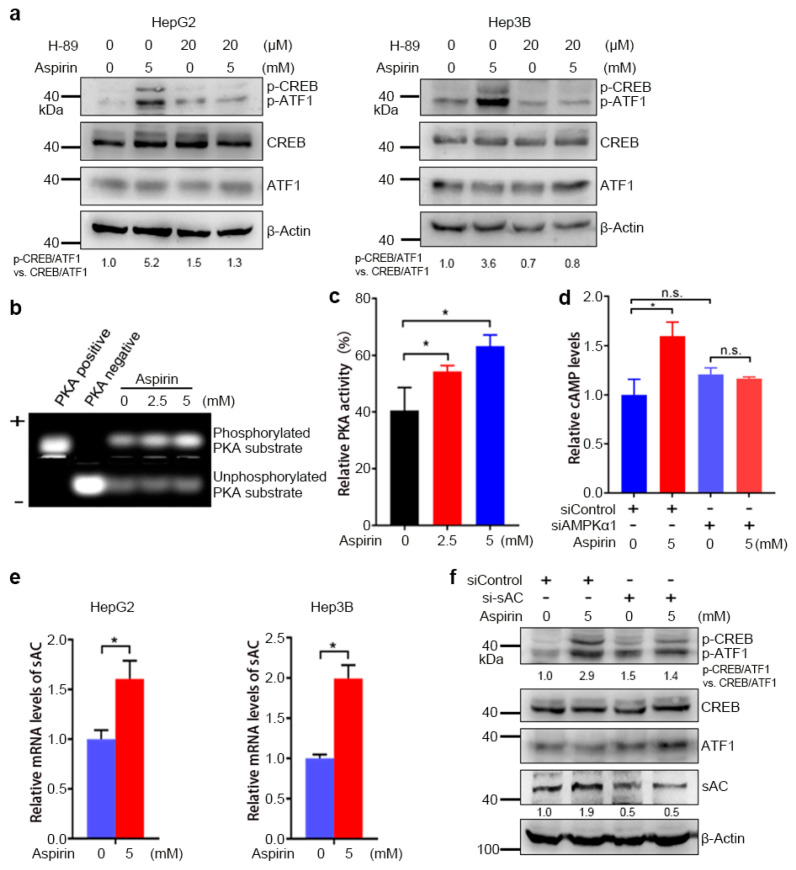
Aspirin activates the sAC–cAMP–PKA–CREB/ATF1 pathway through AMPK. (**a**) HepG2 and Hep3B cells were incubated with H-89 and/or aspirin for 32 h, followed by western blot analysis with indicated antibodies. The relative levels of p-CREB/ATF1 normalized to total CREB/ATF1 were shown. (**b**) HepG2 cells were treated with aspirin for 48 h, followed by PKA kinase assay. The phosphorylated and unphosphorylated PKA substrate kemptide were separated in agarose gel. The positive and negative controls were shown. (**c**) The phosphorylated band in ***b*** was cut and dissolved, followed by detecting the fluorescence values. The PKA activity in the PKA-positive group was set as 100%. The PKA activity in aspirin-treated and vehicle-treated groups was plotted. Values represent means ± SD (*n* = 3). *, *p* < 0.05. (**d**) HepG2 cells were transfected with siControl or siAMPKα1 and treated with or without aspirin for 1 h, followed by cAMP assays. The relative cAMP levels were plotted. Values represent means ± SD (*n* = 3). *, *p* < 0.05. n.s., not significant. (**e**) HepG2 and Hep3B cells were treated with aspirin for 48 h, followed by real-time RT–PCR analysis of sAC transcription. The relative mRNA levels were plotted. Values represent means ± SD (*n* = 3). *, *p* < 0.05. (**f**) HepG2 cells were transfected with siControl or si-sAC and treated with or without aspirin for 32 h, followed by Western blot analysis with indicated antibodies. The relative levels of p-CREB/ATF1 normalized to total CREB/ATF1, and relative levels of sAC normalized to β-actin were shown.

The CREB family transcription factors can be activated by multiple kinases, including Akt, Erk, PKA, p38 and calmodulin kinase (CAMK), some of which may be indirectly activated by AMPK [37]. To determine the kinases that may be involved in the induction of CREB/ATF1 phosphorylation by aspirin, HepG2 cells were treated with or without inhibitors of Akt, MEK, PKA, p38 and CAMK, followed by Western blot analysis of CREB/ATF1 phosphorylation. Among these kinases inhibitors, the PKA inhibitor H-89 and the p38 inhibitor SB203580 abrogated the induction of CREB/ATF1 phosphorylation by aspirin (Figure S3). Meanwhile, H-89 abrogated the induction of CREB/ATF1 phosphorylation by the AMPK agonist AICAR (Figure S4). These data indicate that PKA may act downstream of AMPK to mediate the induction of CREB/ATF1 phosphorylation by aspirin. Next, we confirmed that the PKA inhibitor H89 abrogated the induction of CREB/ATF1 phosphorylation by aspirin in both HepG2 and Hep3B cells (Figure 2a). To determine aspirin’s effect on PKA activity, HepG2 cells were treated with or without aspirin, followed by detection of PKA activity. Aspirin enhanced PKA activity in a dose-dependent manner (Figure 2b,c). Given that PKA is activated by cAMP, we then detected if aspirin increased cAMP levels in HCC cells. Indeed, treatment of HepG2 cells with aspirin led to an increase in cAMP levels (Figure 2d). Knockdown of AMPKα1 abrogated the induction of cAMP by aspirin (Figure 2d). Collectively, these data indicate that AMPK mediates the induction of cAMP synthesis and PKA activation by aspirin.

Soluble adenylyl cyclase (sAC) is one of the enzymes that catalyze cAMP synthesis [34]. We then detected whether the induction of CREB/ATF1 phosphorylation by aspirin is dependent on sAC. Treatment of HepG2 cells with aspirin led to increased sAC expression in both HepG2 and Hep3B cells (Figure 2e). Furthermore, sAC knockdown compromised the induction of CREB/ATF1 phosphorylation by aspirin (Figure 2f). Together, these data indicate that the induction of CREB/ATF1 phosphorylation by aspirin is dependent on sAC.

### 3.3. Aspirin Downregulates CPS1 and Increases cAMP Synthesis in HCC Cells

**Figure 3:**
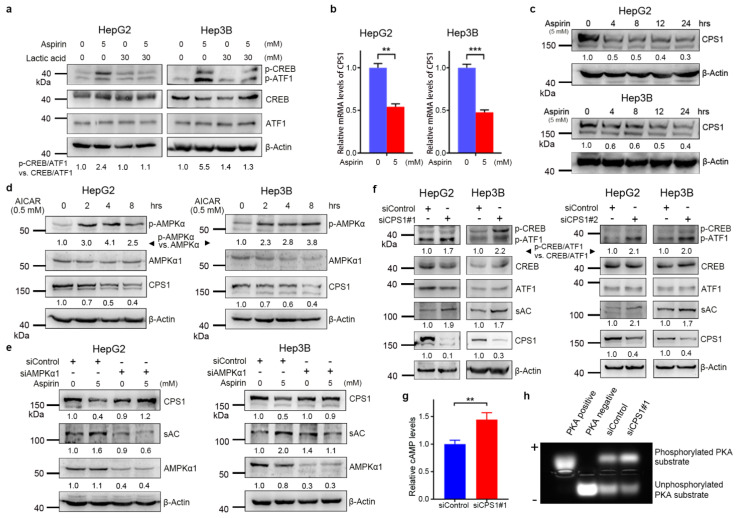
Aspirin inhibits carbamoyl phosphate synthase 1 (CPS1) expression through AMPK activation, and CPS1 knockdown leads to increased cAMP levels, PKA activity and CREB/ATF1 phosphorylation in hepatocellular carcinoma (HCC) cells. (**a**) HepG2 and Hep3B cells were incubated with or without aspirin. Thirty hours later, the cells were treated with lactic acid for another 2 h, followed by Western blot analysis of indicated proteins. The relative levels of p-CREB/ATF1 normalized to total CREB/ATF1 were shown. (**b**) HepG2 and Hep3B cells were treated with or without aspirin for 48 h, followed by real-time RT–PCR analysis of CPS1 transcription. The relative mRNA levels were plotted. Values represent means ± SD (*n* = 3). **, *p* < 0.01, ***, *p* < 0.001. (**c**) HepG2 and Hep3B cells were treated with aspirin for indicated hours, followed by Western blot analysis of CPS1 expression. The relative levels of CPS1 were shown. (**d**) HepG2 and Hep3B cells were treated with AICAR for indicated hours, followed by Western blot analysis of indicated proteins. The relative levels of p-CREB/ATF1 normalized to total CREB/ATF1, and the relative levels of CPS1 normalized to β-actin were shown. (**e**) HepG2 and Hep3B cells were transfected with siControl or siAMPKα1 and treated with or without aspirin for 48 h, followed by Western blot analysis of indicated proteins. The relative levels of CPS1, sAC and AMPKα1 normalized to β-actin were shown. (**f**) HepG2 and Hep3B cells were transfected with siControl or two different siRNA duplexes against CPS1 (siCPS1#1, siCPS1#2) for 48 h, followed by Western blot analysis with indicated antibodies. The relative levels of p-CREB/ATF1 normalized to total CREB/ATF1, and the relative levels of CPS1, sAC normalized to β-actin were shown. (**g**) HepG2 cells were transfected with siControl or siCPS1#1 for 48 h, followed by cAMP assays. The relative cAMP levels were plotted. Values represent means ± SD (*n* = 3). **, *p* < 0.01. (**h**) HepG2 cells were transfected with siControl or siCPS1#1 for 48 h, followed by PKA kinase assay. PKA-phosphorylated substrate migrated toward the anode (+), whereas the unphosphorylated substrate migrated toward the cathode (-).

The expression and activation of sAC are regulated by intracellular bicarbonate levels (HCO_3_^−^) [38]. We found that treatment with lactic acid, the HCO_3_^−^ neutralizer, abrogated the induction of CREB/ATF1 phosphorylation by aspirin in both HepG2 and Hep3B cells (Figure 3a), suggesting that bicarbonate may mediate the induction of the sAC–cAMP–CREB axis by aspirin. It is known that carbonic anhydrase (CA) is responsible for the production of bicarbonate. However, aspirin did not affect the expression of the tumor-associated CA2, CA9 and CA12 (Figure S5). Of note, the urea cycle enzyme CPS1 catalyzes the condensation of ammonia with bicarbonate into carbamoyl phosphate. Hence, CPS1 may deplete both ammonia and bicarbonate. We then detected the effect of aspirin on CPS1. Treatment of HepG2 and Hep3B cells with aspirin led to decreased CPS1 expression at both mRNA and protein levels (Figure 3b,c). A previous study demonstrated that AMPK inactivation might lead to an increase in FOXA1-dependent CPS1 transcription in KRAS/LKB1-mutant NSCLC cells [27]. We then detected the effect of AMPK on CPS1 expression in HCC cells. The AMPK agonist AICAR inhibited CPS1 expression in both HepG2 and Hep3B cells (Figure 3d). AMPK knockdown abrogated the downregulation of CPS1 and upregulation of sAC by aspirin (Figure 3e), suggesting that aspirin inhibited CPS1 expression and promoted sAC expression through AMPK. Except for bicarbonate accumulation, a rise in intracellular ammonia levels may result from decreased CPS1 expression. Treatment of HepG2 cells with the ammonia donor NH_4_Cl did not induce CREB/ATF1 phosphorylation, suggesting that ammonia is not attributable to CREB/ATF1 phosphorylation (Figure S6).

We further determined whether CPS1 regulated the sAC–cAMP–PKA–CREB pathway. CPS1 knockdown led to an increase in sAC expression and CREB/ATF1 phosphorylation in both HepG2 and Hep3B cells (Figure 3f). In addition, CPS1 knockdown led to an increase in the levels of cAMP and PKA activity (Figure 3g,h). These data suggest that CPS1 is a negative regulator of the cAMP–PKA–CREB/ATF1 signaling axis.

### 3.4. CREB/ATF1 Knockdown Sensitizes HCC Cells to Aspirin

**Figure 4:**
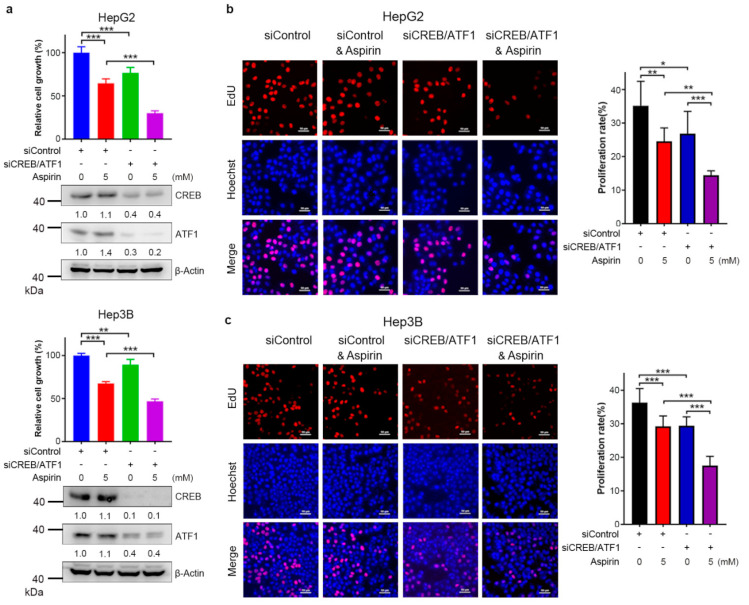
CREB/ATF1 knockdown sensitizes HCC cells to aspirin. (**a**) HepG2 and Hep3B cells were transfected with siControl or siCREB/ATF1 and treated with or without aspirin for 48 h, followed by CCK-8 assays. The relative cell growth was plotted. Values represent means ± SD (*n* = 5). ***, *p* < 0.001. The efficiency of CREB and ATF1 knockdown was detected by Western blotting. (**b**,**c**) HepG2 and Hep3B cells were transfected with siControl or siCREB/ATF1 and treated with or without aspirin for 48 h, followed by detecting proliferative cells with EdU assays. In addition, all cells were labeled with Hoechst33258. The proliferation rate was plotted. Values represent means ± SD (*n* = 3). *, *p* < 0.05, **, *p* < 0.01, ***, *p* < 0.001.

To determine the effect of CREB/ATF1 on the sensitivity of HCC cells to aspirin, HepG2 and Hep3B cells were transfected with siControl or siCREB/ATF1, followed by treatment with or without aspirin. Forty-eight hours later, cell growth was detected by CCK-8 assays. CREB/ATF1 knockdown sensitized HepG2 and Hep3B cells to aspirin (Figure 4a). In addition, EdU assays demonstrated that aspirin in combination with CREB/ATF1 knockdown significantly inhibited both HepG2 and Hep3B cell proliferation (Figure 4b,c). These data suggest that CREB/ATF1 activation may antagonize the anti-cancer effect of aspirin, and targeting CREB/ATF1 may be a strategy to sensitize HCC cells to aspirin.

### 3.5. The Natural Agent Berbamine Inhibits CREB/ATF1 Phosphorylation and Sensitizes HCC Cells to Aspirin

**Figure 5:**
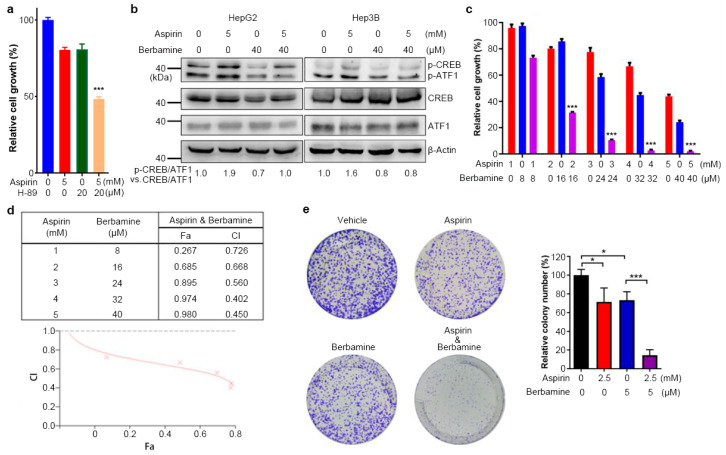
Berbamine inhibits the induction of CREB/ATF1 phosphorylation by aspirin and synergizes with aspirin to inhibit HCC cell growth. (**a**) HepG2 cells were treated with aspirin alone or in combination with H-89 for 48 h, followed by CCK-8 assays. The relative cell growth was plotted. Values represent means ± SD (*n* = 5). ***, *p* < 0.001, compared to other groups. (**b**) HepG2 and Hep3B cells were treated with aspirin individually or in combination with berbamine for 32 h, followed by Western blot analysis of indicated proteins. The relative levels of p-CREB/ATF1 normalized to total CREB/ATF1 were shown. (**c**) HepG2 cells were treated with aspirin alone or in combination with berbamine for 48 h, followed by CCK-8 assays. The relative cell growth was plotted. The cell viability in aspirin- and berbamine-untreated group was set as 100%. Values represent means ± SD (*n* = 5). ***, *p* < 0.001, compared to mono-treatment. (**d**) The combination index (CI) was identified using CalcuSyn software and shown by the Fa-CI plot. (**e**) HepG2 cells were treated with aspirin and/or berbamine for 48 h. The cells were incubated for another 7 days, followed by crystal violet staining. The relative colony formation was plotted. Values represent means ± SD (*n* = 3). *, *p* < 0.05. ***, *p* < 0.001.

Aspirin, in combination with other treatments, may achieve a more significant anti-cancer effect for established tumors [9,14]. Abrogation of the induction of CREB/ATF1 phosphorylation by aspirin may be a strategy to improve the anti-HCC effect of aspirin. Indeed, the combination of aspirin and the PKA inhibitor H-89 more significantly inhibited HepG2 cell growth than the single-agent (Figure 5a). Since H-89 is not a good candidate for clinical use, we then searched other drugs that might block CREB/ATF1 and synergize with aspirin to inhibit HCC. We found that berbamine, a natural agent that has been used as a traditional medicine in clinics to ameliorate leukopenia caused by cancer radiotherapy and chemotherapy [39], could inhibit the induction of CREB/ATF1 phosphorylation by aspirin in HepG2 and Hep3B cells (Figure 5b). Next, we determined the effect of combining aspirin and berbamine on HepG2 cell growth. Indeed, berbamine synergized with aspirin to inhibit HepG2 cell growth (Figure 5c,d). All combination index (CI) values were less than 0.9, and the CI values were less than 0.5 at certain concentrations, indicating that the effect of aspirin and berbamine in HepG2 cell growth is synergistic rather than additive (Figure 5d). Meanwhile, the combination of aspirin and berbamine significantly inhibited HepG2 cell colony formation than each agent alone (Figure 5e). Together, these data demonstrate that aspirin and berbamine synergistically inhibit HCC cell growth. While berbamine is also a CAMK2G inhibitor [35], CAMK2G knockdown neither sensitized Hep3B cells to aspirin nor did it abrogate the induction of CREB/ATF1 phosphorylation (Figure S7), indicating that CAMK2G inhibition is less likely attributable to the synergism between aspirin and berbamine.

**Figure 6:**
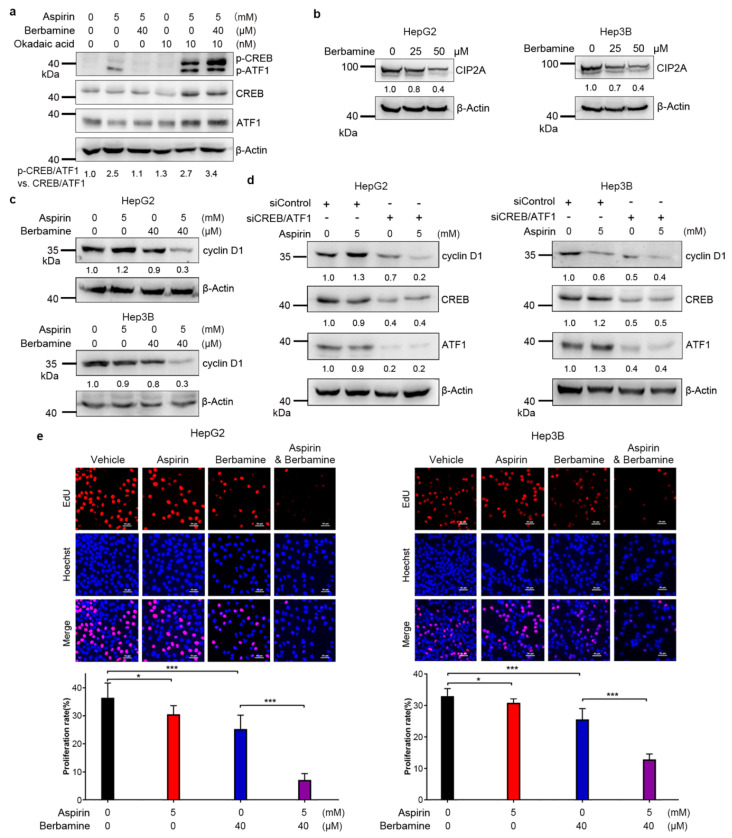
Berbamine inhibits protein phosphatase 2A (CIP2A) expression, promotes PP2A-mediated CREB/ATF1 dephosphorylation, and potentiates the inhibition of cyclin D1 expression in HCC cells. (**a**) HepG2 cells were treated with or without aspirin, berbamine and okadaic acid, followed by western blot analysis of CREB/ATF1 phosphorylation. The relative levels of p-CREB/ATF1 normalized to total CREB/ATF1 were shown. (**b**) HepG2 and Hep3B cells were treated with indicated doses of berbamine, followed by western blot analysis of CIP2A. The relative levels of CIP2A were shown. (**c**) HepG2 and Hep3B cells were treated with aspirin and/or berbamine for 48 h, followed by western blot analysis of cyclin D1 expression. The relative levels of cyclin D1 were shown. (**d**) HepG2 and Hep3B cells were transfected with siControl or siCREB/ATF1 and treated with or without aspirin for 48 h, followed by western blot analysis of indicated proteins. The relative levels of cyclin D1, CREB and ATF1 were shown. (**e**) HepG2 and Hep3B cells were treated with aspirin individually or in combination with berbamine for 48 h, followed by detecting proliferative cells with EdU assays. *Scale bar,* 50 μm. The proliferation rate was plotted. Values represent means ± SD (*n* = 3). ***, *p* < 0.001.

Next, we investigated the mechanisms underlying the inhibition of CREB/ATF1 phosphorylation by berbamine. While berbamine did not inhibit PKA activity (Figure S8), treatment with the protein phosphatase 2A (PP2A) inhibitor okadaic acid abrogated the inhibition of aspirin-induced CREB/ATF1 phosphorylation by berbamine, indicating that PP2A may mediate the inhibition of CREB/ATF1 phosphorylation by berbamine (Figure 6a). Given that the cancerous inhibitor of PP2A (CIP2A) is an endogenous PP2A inhibitor [40], we detected the effect of berbamine on CIP2A expression. Treatment with berbamine inhibited the expression of CIP2A in both HepG2 and Hep3B cells (Figure 6b).

Aspirin slightly increased the expression of cyclin D1, a known CREB/ATF1 target, in HepG2 cells (Figure 6c,d). However, the combination of aspirin and berbamine dramatically inhibited the expression of cyclin D1 in HepG2 cells (Figure 6c). In addition, the combination of aspirin and berbamine significantly inhibited cyclin D1 expression in Hep3B cells (Figure 6c). Moreover, CREB/ATF1 knockdown inhibited cyclin D1 expression in both HepG2 and Hep3B cells (Figure 6d). The combination of CREB/ATF1 knockdown and aspirin more significantly inhibited cyclin D1 expression in HepG2 cells than in each treatment alone (Figure 6d). Together, these data indicate that activation of CREB/ATF1 may antagonize the ability of aspirin to inhibit cyclin D1 expression. Thus, inhibition of CREB/ATF1 could potentiate the inhibition of cyclin D1 by aspirin. Furthermore, the percentage of EdU-labeled HepG2 and Hep3B cells was more dramatically reduced by cotreatment with aspirin and berbamine compared to each agent alone (Figure 6e), indicating that combination of aspirin and berbamine more strongly inhibited S-phase entry and cell proliferation. No obvious cell death was observed in aspirin- and berbamine-treated HepG2 and Hep3B cells. Treatment of HepG2 cells with the pan-caspase inhibitor and necroptosis inhibitor had no effects on the inhibition of cell growth by combined treatment with aspirin and berbamine (Figure S9).

### 3.6. Combination of Aspirin and Berbamine Synergistically Inhibits HCC Growth

**Figure 7:**
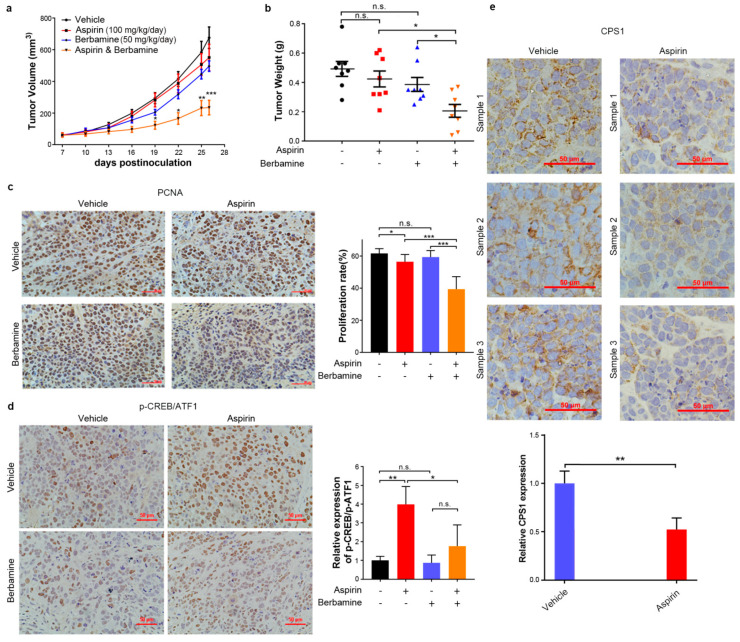
Combined treatment with aspirin and berbamine inhibits HepG2 xenograft tumors in nude mice. (**a**) BALB/c-nu mice with HepG2 xenografts were treated with or without aspirin and/or berbamine. The tumor growth curves were plotted. Values represent means ± SD (*n* = 8). *, *p* < 0.05, **, *p* < 0.01, ***, *p* < 0.001, compared with tumors treated with berbamine alone. (**b**) The tumor weight was plotted. Values represent means ± SD (*n* = 8). *, *p* < 0.05. n.s., not significant. (**c**) Immunohistochemical staining of PCNA in tumor tissue sections. The proliferation rate was plotted. *, *p* < 0.05, ***, *p* < 0.001. (**d**) Immunohistochemical analysis of CREB/ATF1 phosphorylation in tumor tissue sections. The relative CREB/ATF1 phosphorylation was plotted. *n.s.*, not significant. *, *p* < 0.05, **, *p* < 0.01. (**e**) Immunohistochemical staining of CPS1 in tumor tissue sections. Representative immunohistochemical staining of vehicle- or aspirin-treated tumors was shown. *Scale bar*, 50 μm. The relative CPS1 expression was plotted. **, *p* < 0.01.

The tumor-suppressive effect of aspirin in combination with berbamine was detected in the HepG2 xenografts model. While aspirin- or berbamine-treated tumors tended to be smaller than vehicle (control)-treated tumors, this difference was not statistically significant. The combination of aspirin and berbamine more significantly inhibited tumor growth compared to the single-agent (Figure 7a,b). Immunohistochemical analysis of PCNA in tumor samples demonstrated that the combination of aspirin and berbamine more significantly inhibited cancer cell proliferation than single-agent (Figure 7c). Consistent with the in vitro data, treatment with aspirin led to increased CREB/ATF1 phosphorylation in tumors, which was abrogated by berbamine (Figure 7d). Whereas aspirin downregulates CPS1 via inducing AMPK activation in HCC cells, a previous study demonstrates that aspirin does not induce AMPK phosphorylation in the normal liver [15]. We then detected the effects of aspirin on CPS1 expression in liver tissues and xenograft tumors. Whereas aspirin inhibited CPS1 expression in HCC xenografts (Figure 7e), it did not affect CPS1 expression in liver tissues (Figure S10). Meanwhile, aspirin did not induce CREB/ATF1 phosphorylation in the normal liver tissues (Figure S10).

## 4. Discussion

Aspirin is an effective chemopreventive agent for some types of cancer, including colorectal and liver cancer. Although aspirin monotherapy has a limited effect on established cancer, aspirin can enhance the effect of neoadjuvant chemoradiotherapy and improve the prognosis of patients with rectal cancer [41]. A better understanding of the effects of aspirin on cell signaling may help maximize its anti-cancer effects. Here, we show that aspirin induces PKA activation and CREB/ATF1 phosphorylation in HCC cells. Furthermore, inhibition of PKA or CREB/ATF1 sensitizes HCC cells to aspirin. Combination of aspirin and berbamine, a natural product that inhibits CREB/ATF1 phosphorylation, achieves a significant therapeutic effect on HCC in a murine model. Our results support the concepts that aspirin has multiple effects on signaling pathways in cancer cells and that inhibiting some of these signaling pathways may enhance aspirin’s anti-cancer effects.

Previous studies have demonstrated that aspirin can directly or indirectly activate AMPK, a contextual tumor suppressor or promoter [9,12,13]. AMPK can inhibit mTORC1 through suppressing raptor or phosphorylating TSC2, a negative regulator of mTORC1 [22,23,42]. On the other hand, AMPK can promote lysosome biogenesis via TFE3 or BRD4 and protect cancer cells under stresses [43]. In addition, AMPK may directly or indirectly phosphorylate CREB at S133 [44]. Our data indicate that the induction of CREB/ATF1 phosphorylation by aspirin in HCC cells is predominantly mediated by the indirect activation of PKA by AMPK. The COX-PGE2-EP2 signaling axis also upregulates the cAMP–PKA pathway [36]. As an AMPK activator and a COX inhibitor, aspirin may regulate PKA activity in two opposing directions. The upregulation of PKA activity in HCC cells by aspirin indicates that AMPK-mediated PKA activation outweighs PKA inactivation resulting from COX inhibition if the latter situation does happen. CREB has been identified to promote tumor progression and to be associated with the overall survival and therapy response of patients with many types of cancer, while it may be tumor-suppressive in some types of cancer [45,46,47]. From a therapeutic perspective, the induction of PKA and CREB/ATF1 activation by aspirin and AMPK may be a disadvantage for treating many cancer types. In fact, our results show that inhibition of PKA and CREB/ATF1 can improve the anti-HCC effects of aspirin.

An important finding of the current study is that aspirin can inhibit the urea cycle enzyme CPS1 expression in HCC cells, an effect also related to AMPK. While AMPK reportedly inhibits CPS1 expression in KRAS-mutant lung cancer cells [27], our results show that it also inhibits CPS1 expression in HCC cells without KRAS mutation. Similar to AMPK, CPS1 has contrasting effects on tumorigenesis. CPS1 depletion may result in decreased levels of the metabolic intermediate carbamoyl phosphate and impaired NSCLC cell survival [27,48]. In contrast, *CPS1* downregulation is thought to promote hepatocarcinogenesis through increased glutamine availability for de novo pyrimidine biosynthesis [28,32].

CPS1 depletion is supposed to lead to ammonia accumulation. Although ammonia is usually considered as a highly toxic waste product of glutaminolysis reaction, it can be effectively incorporated into amino acids, including glutamate, proline, aspartate and alanine in breast cancer cells [30]. While tumor cells with CPS1 overexpression generate high amounts of carbamoyl phosphate that may translocate into the cytoplasm and then support CAD (carbamoyl-phosphate synthetase 2, aspartate transcarbamylase, and dihydroorotase)-mediated de novo pyrimidine synthesis [27,31,48], the reduction of CPS1 in HCC rewires ammonia metabolism by recycling ammonia to glutamine for the initiation of de novo pyrimidine biosynthesis [28,49]. Given that the activity of CAD is positively regulated by the mTOR-S6K1 pathway [50], and aspirin can inhibit mTOR through activation of AMPK, it is less likely that aspirin may paradoxically promote CAD-mediated pyrimidine synthesis as a result of CPS1 downregulation.

Hence, far, little is known about the role of CPS1 in cell signaling. The current study demonstrates that depletion of CPS1 leads to increased CREB/ATF1 phosphorylation in HCC cells, which may represent a noncanonical function of CPS1. While CPS1 catalyzes the production of carbamoyl phosphate, it consumes bicarbonate and ammonia. Therefore, CPS1 loss may reduce bicarbonate depletion, a pH regulator, metabolic intermediate and signal transducer. sAC is directly stimulated by bicarbonate to produce the PKA activator cAMP. AMPK-mediated downregulation of CPS1 and subsequent activation of sAC and PKA may contribute, at least in part, to the induction of CREB/ATF1 phosphorylation by aspirin. The diversion of bicarbonate to support cAMP synthesis and PKA–CREB signaling by aspirin-induced CPS1 depletion may counteract aspirin’s detrimental effects on HCC cells. Thus, targeting the PKA/CREB pathway is a strategy for maximizing the anti-cancer effects of aspirin. Although aspirin can induce AMPK phosphorylation in colon tissues, it does not induce AMPK phosphorylation in the normal liver [13]. Consistent with these data, we find that aspirin neither inhibits CPS1 expression nor induces CREB/ATF1 phosphorylation in normal liver. Therefore, it is less likely that aspirin may impair ammonia detoxification in the liver when it is used as a chemopreventive or therapeutic agent.

Our current study demonstrates that berbamine can inhibit CIP2A expression and abrogate the induction of CREB/ATF1 phosphorylation by aspirin in a PP2A-dependent manner. Given that STAT3 upregulates CIP2A expression, and berbamine is a STAT3 inhibitor [51,52], the inhibition of CIP2A expression by berbamine may result from STAT3 inhibition. Cyclin D1 is one of CREB/ATF1 and wnt/β-catenin targets and a critical driver of cancer cell proliferation. On one hand, aspirin may inhibit cyclin D1 expression by suppressing wnt/β-catenin signaling. On the other hand, the induction of CREB/ATF1 activation by aspirin may enhance cyclin D1 expression. The balance between these two opposing effects may dictate whether aspirin inhibits or promotes cyclin D1 expression in HCC cells. Hence, inhibition of CREB/ATF1 activation is supposed to potentiate the suppression of cyclin D1 expression by aspirin. In addition, aspirin induces the expression of other CREB/ATF1-responsive genes, such as c-Jun, in AMPK-dependent manner. Except for suppression of cyclin D1 expression, the abrogation of aspirin-induced c-Jun expression by CREB/ATF1 inhibition may also contribute to the synergistic inhibition of HCC cells growth by CREB/ATF1 inactivation and aspirin.

**Figure 8:**
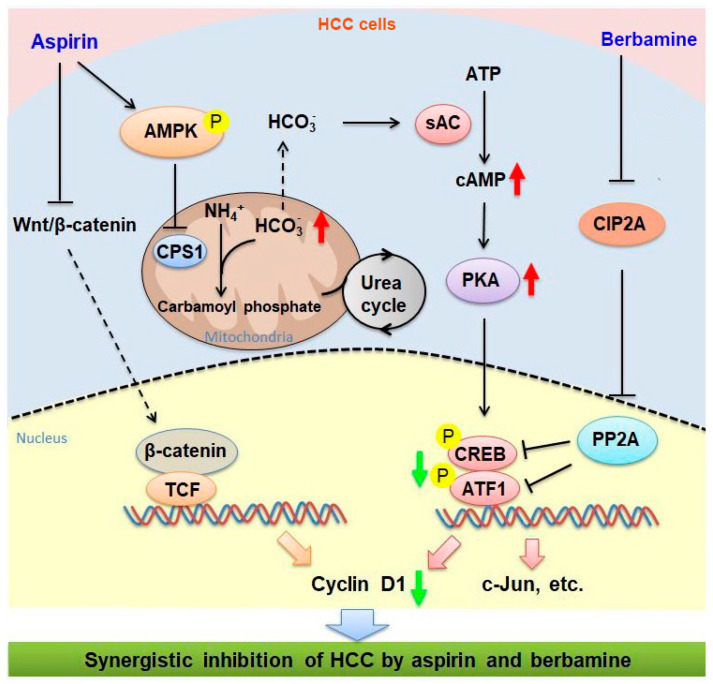
Schematic illustration of the signaling pathways that are involved in the synergistic inhibition of HCC by aspirin and berbamine. While aspirin may inhibit Wnt/β-catenin signaling, it can activate AMPK, leading to downregulation of CPS1 in HCC cells. Downregulation of CPS1 diverts bicarbonate to stimulate sAC, thereby increasing cAMP synthesis, which may contribute, at least in part, to the stimulation of PKA activation and CREB/ATF1 phosphorylation in HCC cells by aspirin. Berbamine can inhibit CIP2A expression in HCC cells, leading to PP2A-dependent abrogation of aspirin-induced CREB/ATF1 phosphorylation and target gene expression. Thus, the combination of aspirin and berbamine can synergistically inhibit HCC. Red arrows represent the upregulation of indicated molecules by aspirin. Green arrows represent the net effects of combined treatment with aspirin and berbamine on indicated proteins.

Combination treatment is a particularly important strategy to combat cancer [53]. While aspirin monotherapy is less effective in HCC models, the combination of aspirin and sorafenib can significantly inhibit HCC growth [8,9]. Our current study demonstrates that berbamine can synergize with aspirin to inhibit HCC cells. The mechanisms underlying the synergistic inhibition of HCC by aspirin and berbamine are illustrated in Figure 8. Notably, berbamine has been administered in the clinic to prevent leukopenia caused by cancer radiotherapy and chemotherapy [39]. Both aspirin and berbamine are inexpensive and easily accessible drugs. The combination of berbamine and aspirin may be a feasible treatment for many HCC patients. Clinical trials are warranted to determine the efficacy of this regimen in patients with HCC. Except for systemic treatment with aspirin and berbamine, aspirin combined with berbamine may be a topical use during downstaging procedures for advanced HCC, which awaits further study. Meanwhile, it remains to know whether combined treatment with aspirin and berbamine is effective for non-HCC malignancy in the liver, such as intrahepatic cholangiocarcinoma.

## 5. Conclusions

In summary, the current study demonstrates that CPS1 can regulate cAMP–PKA–CREB/ATF1 signaling. The AMPK-mediated downregulation of CPS1 and subsequent upregulation of cAMP–PKA–CREB/ATF1 signaling restricts the anti-HCC effects of aspirin. Inhibition of PKA and CREB/ATF1 activation represents a novel strategy to potentiate the anti-HCC effects of aspirin. The combination of aspirin with the natural agent berbamine holds promise in HCC prevention and treatment. The data in this study could be instrumental in identifying signaling pathways involved in aspirin resistance, which can be therapeutically targeted for boosting the anti-cancer potential of aspirin.

## Data Availability

The data for the current study are available from the corresponding author on reasonable request.

## Supplementary Materials

The following are available online at https://www.mdpi.com/article/10.3390/cancers13071738/s1, Figure S1: The AMPK agonist AICAR induces CREB/ATF1 phosphorylation in HepG2 cells. Figure S2: AMPKα1 knockdown abrogates the induction of c-Jun transcription by aspirin. Figure S3: The effects of MEK inhibitor U0126, Akt inhibitor MK2206, PKA inhibitor H-89, CAMK inhibitor KN93 and p38 inhibitor SB203580 on the induction of CREB/ATF1 phosphorylation by aspirin in HepG2 cells. Figure S4: The effects of PKA inhibitor H-89 on the induction of CREB/ATF1 phosphorylation by AMPK agonist AICAR. Figure S5: The effects of aspirin on CA2, CA9 and CA12 expression in HepG2 cells. Figure S6: NH_4_Cl does not induce CREB/ATF1 phosphorylation in HepG2 cells. Figure S7: CAMK2G knockdown does not sensitize Hep3B cells to aspirin. Figure S8: Berbamine does not inhibit PKA activity. HepG2 cells were treated with or without berbamine, followed by PKA kinase assay. Figure S9: The caspase inhibitor Z-VAD-FMK and necroptosis inhibitor necrostatin-1 do not affect the inhibition of HepG2 cell growth by aspirin and berbamine. Figure S10: Aspirin does not induce CREB/ATF1 phosphorylation and inhibits CPS1 expression in mouse liver tissues. Table S1: List of the chemicals used in this study. Table S2: List of the antibodies used in this study. Table S3: List of siRNA sequences for RNA interference. Table S4: List of the primer sequences for quantitative reverse-transcription polymerase chain reaction. File S1: The original Western blots.

## References

[1] Hua H., Zhang H., Kong Q., Wang J., Jiang Y. (2019). Complex roles of the old drug aspirin in cancer chemoprevention and therapy. Med. Res. Rev..

[2] Simon T.G., Ma Y., Ludvigsson J.F., Chong D.Q., Giovannucci E.L., Fuchs C.S., Meyerhardt J.A., Corey K.E., Chung R.T., Zhang X. (2018). Association between aspirin use and risk of hepatocellular carcinoma. JAMA Oncol..

[3] Sitia G., Aiolfi R., Di Lucia P., Mainetti M., Fiocchi A., Mingozzi F., Esposito A., Ruggeri Z.M., Chisari F.V., Iannacone M. (2012). Antiplatelet therapy prevents hepatocellular carcinoma and improves survival in a mouse model of chronic hepatitis B. Proc. Natl. Acad. Sci. USA.

[4] Simon T.G., Henson J., Osganian S., Masia R., Chan A.T., Chung R.T., Corey K.E. (2019). Daily aspirin use associated with reduced risk for fibrosis progression in patients with nonalcoholic fatty liver disease. Clin. Gastroenterol. Hepatol..

[5] Lee T.Y., Hsu Y.C., Tseng H.C., Yu S.H., Lin J.T., Wu M.S., Wu C.Y. (2019). Association of daily aspirin therapy with risk of hepatocellular carcinomain patients with chronic hepatitis B. JAMA Intern. Med..

[6] Liao Y.H., Hsu R.J., Wang T.H., Wu C.T., Huang S.Y., Hsu C.Y., Su Y.C., Hsu W.L., Liu D.W. (2020). Aspirin decreases hepatocellular carcinoma risk in hepatitis C virus carriers: A nationwide cohort study. BMC Gastroenterol..

[7] Li S., Dai W., Mo W., Li J., Feng J., Wu L., Liu T., Yu Q., Xu S., Wang W. (2017). By inhibiting PFKFB3, aspirin overcomes sorafenib resistance in hepatocellular carcinoma. Int. J. Cancer.

[8] Hammerlindl H., Ravindran Menon D., Hammerlindl S., Emran A.A., Torrano J., Sproesser K., Thakkar D., Xiao M., Atkinson V.G., Gabrielli B. (2018). Acetylsalicylic acid governs the effect of sorafenib in RAS-mutant cancers. Clin. Cancer Res..

[9] Gao M., Kong Q., Hua H., Yin Y., Wang J., Luo T., Jiang Y. (2016). AMPK-mediated up-regulation of mTORC2 and MCL-1 compromises the anti-cancer effects of aspirin. Oncotarget.

[10] Lu L., Sun H.C., Zhang W., Chai Z.T., Zhu X.D., Kong L.Q., Wang W.Q., Zhang K.Z., Zhang Y.Y., Zhang Q.B. (2013). Aspirin minimized the pro-metastasis effect of sorafenib and improved survival by up-regulating HTATIP2 in hepatocellular carcinoma. PLoS ONE.

[11] Ornelas A., Zacharias-Millward N., Menter D.G., Davis J.S., Lichtenberger L., Hawke D., Hawk E., Vilar E., Bhattacharya P., Millward S. (2017). Beyond COX-1: The effects of aspirin on platelet biology and potential mechanisms of chemoprevention. Cancer Metastasis Rev..

[12] Hawley S.A., Fullerton M.D., Ross F.A., Schertzer J.D., Chevtzoff C., Walker K.J., Peggie M.W., Zibrova D., Green K.A., Mustard K.J. (2012). The ancient drug salicylate directly activates AMP-activated protein kinase. Science.

[13] Din F.V., Valanciute A., Houde V.P., Zibrova D., Green K.A., Sakamoto K., Alessi D.R., Dunlop M.G. (2012). Aspirin inhibits mTOR signaling, activates AMP-activated protein kinase, and induces autophagy in colorectal cancer cells. Gastroenterology.

[14] Henry W.S., Laszewski T., Tsang T., Beca F., Beck A.H., McAllister S.S., Toker A. (2017). Aspirin suppresses growth in PI3K-mutant breast cancer by activating AMPK and inhibiting mTORC1 signaling. Cancer Res..

[15] Lucido M.J., Orlando B.J., Vecchio A.J., Malkowski M.G. (2016). Crystal structure of aspirin-acetylated human cyclooxygenase-2: Insight into the formation of products with reversed stereochemistry. Biochemistry.

[16] Passacquale G., Phinikaridou A., Warboys C., Cooper M., Lavin B., Alfieri A., Andia M.E., Botnar R.M., Ferro A. (2015). Aspirin-induced histone acetylation in endothelial cells enhances synthesis of the secreted isoform of netrin-1 thus inhibiting monocyte vascular infiltration. Br. J. Pharmacol..

[17] Pietrocola F., Castoldi F., Markaki M., Lachkar S., Chen G., Enot D.P., Durand S., Bossut N., Tong M., Malik S.A. (2018). Aspirin recapitulates features of caloric restriction. Cell Rep..

[18] Clement S.T., Dixit G., Dohlman H.G. (2013). Regulation of yeast G protein signaling by the kinases that activate the AMPK homolog Snf1. Sci. Signal..

[19] Yuan J., Dong X., Yap J., Hu J. (2020). The MAPK and AMPK signalings: Interplay and implication in targeted cancer therapy. J. Hematol. Oncol..

[20] Hardie D.G., Schaffer B.E., Brunet A. (2016). AMPK: An energy-sensing pathway with multiple inputs and outputs. Trends Cell Biol..

[21] Vara-Ciruelos D., Russell F.M., Hardie D.G. (2019). The strange case of AMPK and cancer: Dr Jekyll or Mr Hyde?. Open Biol..

[22] Inoki K., Zhu T., Guan K.L. (2003). TSC2 mediates cellular energy response to control cell growth and survival. Cell.

[23] Gwinn D.M., Shackelford D.B., Egan D.F., Mihaylova M.M., Mery A., Vasquez D.S., Turk B.E., Shaw R.J. (2008). AMPK phosphorylation of raptor mediates a metabolic checkpoint. Mol. Cell.

[24] Jones R.G., Plas D.R., Kubek S., Buzzai M., Mu J., Xu Y., Birnbaum M.J., Thompson C.B. (2005). AMP-activated protein kinase induces a p53-dependent metabolic checkpoint. Mol. Cell.

[25] Jeon S.M., Chandel N.S., Hay N. (2012). AMPK regulates NADPH homeostasis to promote tumour cell survival during energy stress. Nature.

[26] Eichner L.J., Brun S.N., Herzig S., Young N.P., Curtis S.D., Shackelford D.B., Shokhirev M.N., Leblanc M., Vera L.I., Hutchins A. (2019). Genetic analysis reveals AMPK is required to support tumor growth in murine Kras-dependent lung cancer models. Cell Metab..

[27] Kim J., Hu Z., Cai L., Li K., Choi E., Faubert B., Bezwada D., Rodriguez-Canales J., Villalobos P., Lin Y.F. (2017). CPS1 maintains pyrimidine pools and DNA synthesis in KRAS/LKB1-mutant lung cancer cells. Nature.

[28] Keshet R., Szlosarek P., Carracedo A., Erez A. (2018). Rewiring urea cycle metabolism in cancer to support anabolism. Nat. Rev. Cancer.

[29] Altman B.J., Stine Z.E., Dang C.V. (2016). From Krebs to clinic: Glutamine metabolism to cancer therapy. Nat. Rev. Cancer.

[30] Spinelli J.B., Yoon H., Ringel A.E., Jeanfavre S., Clish C.B., Haigis M.C. (2017). Metabolic recycling of ammonia via glutamate dehydrogenase supports breast cancer biomass. Science.

[31] Çeliktas M., Tanaka I., Tripathi S.C., Fahrmann J.F., Aguilar-Bonavides C., Villalobos P., Delgado O., Dhillon D., Dennison J.B., Ostrin E.J. (2017). Role of CPS1 in cell growth, metabolism and prognosis in LKB1-inactivated lung adenocarcinoma. J. Natl. Cancer Inst..

[32] Liu H., Dong H., Robertson K., Liu C. (2011). DNA methylation suppresses expression of the urea cycle enzyme carbamoyl phosphate synthetase 1 (CPS1) in human hepatocellular carcinoma. Am. J. Pathol..

[33] Zhang H., Kong Q., Wang J., Jiang Y., Hua H. (2020). Complex roles of cAMP-PKA-CREB signaling in cancer. Exp. Hematol. Oncol..

[34] Hua H., Kong Q., Yin J., Zhang J., Jiang Y. (2020). Insulin-like growth factor receptor signaling in tumorigenesis and drug resistance: A challenge for cancer therapy. J. Hematol. Oncol..

[35] Meng Z., Li T., Ma X., Wang X., Van Ness C., Gan Y., Zhou H., Tang J., Lou G., Wang Y. (2013). Berbamine inhibits the growth of liver cancer cells and cancer-initiating cells by targeting Ca²⁺/calmodulin-dependent protein kinase II. Mol. Cancer Ther..

[36] Ishihara E., Nagaoka Y., Okuno T., Kofuji S., Ishigami-Yuasa M., Kagechika H., Kamimura K., Terai S., Yokomizo T., Sugimoto Y. (2020). Prostaglandin E2 and its receptor EP2 trigger signaling that contributes to YAP-mediated cell competition. Genes Cells.

[37] Didier S., Sauvé F., Domise M., Buée L., Marinangeli C., Vingtdeux V. (2018). AMP-activated protein kinase controls immediate early genes expression following synaptic activation through the PKA/CREB pathway. Int. J. Mol. Sci..

[38] Sun X.C., Cui M., Bonanno J.A. (2004). [HCO_3_^−^]-regulated expression and activity of soluble adenylyl cyclase in corneal endothelial and Calu-3 cells. BMC Physiol..

[39] Li S.Y., Jei W., Seow W.K., Thong Y.H. (1994). Effect of berbamine on blood and bone-marrow stem cells of cyclophosphamide-treated mice. Int. J. Immunopharmacol..

[40] Soofiyani S.R., Hejazi M.S., Baradaran B. (2017). The role of CIP2A in cancer: A review and update. Biomed. Pharmacother..

[41] Wang B., Huang Y. (2020). Effect of aspirin use on neoadjuvant chemoradiotherapy for rectal cancer: A meta-analysis with trial sequential analysis. J. Cancer Res. Clin. Oncol..

[42] Hua H., Kong Q., Zhang H., Wang J., Luo T., Jiang Y. (2019). Targeting mTOR for cancer therapy. J. Hematol. Oncol..

[43] Sakamaki J.I., Wilkinson S., Hahn M., Tasdemir N., O’Prey J., Clark W., Hedley A., Nixon C., Long J.S., New M. (2017). Bromodomain protein BRD4 is a transcriptional repressor of autophagy and lysosomal function. Mol. Cell.

[44] Thomson D.M., Herway S.T., Fillmore N., Kim H., Brown J.D., Barrow J.R., Winder W.W. (2008). AMP-activated protein kinase phosphorylates transcription factors of the CREB family. J. Appl. Physiol..

[45] Steven A., Friedrich M., Jank P., Heimer N., Budczies J., Denkert C., Seliger B. (2020). What turns CREB on? And off? And why does it matter?. Cell. Mol. Life Sci..

[46] Steven A., Heiduk M., Recktenwald C.V., Hiebl B., Wickenhauser C., Massa C., Seliger B. (2015). Colorectal carcinogenesis: Connecting K-RAS-induced transformation and CREB activity in vitro and in vivo. Mol. Cancer Res..

[47] Friedrich M., Heimer N., Stoehr C., Steven A., Wach S., Taubert H., Hartmann A., Seliger B. (2020). CREB1 is affected by the microRNAs miR-22-3p, miR-26a-5p, miR-27a-3p, and miR-221-3p and correlates with adverse clinicopathological features in renal cell carcinoma. Sci. Rep..

[48] Pham-Danis C., Gehrke S., Danis E., Rozhok A.I., Daniels M.W., Gao D., Collins C., Paola J., D’Alessandro A., DeGregori J. (2019). Urea cycle sustains cellular energetics upon EGFR inhibition in EGFR-mutant NSCLC. Mol. Cancer Res..

[49] Dumenci O.E., Abellona M.R.U., Khan S.A., Holmes E., Taylor-Robinson S.D. (2020). Exploring metabolic consequences of CPS1 and CAD dysregulation in hepatocellular carcinoma by network reconstruction. J. Hepatocell Carcinoma.

[50] Ben-Sahra I., Howell J.J., Asara J.M., Manning B.D. (2013). Stimulation of de novo pyrimidine synthesis by growth signaling through mTOR and S6K1. Science.

[51] Huang Q., Qin S., Yuan X., Zhang L., Ji J., Liu X., Ma W., Zhang Y., Liu P., Sun Z. (2017). Arctigenin inhibits triple-negative breast cancers by targeting CIP2A to reactivate protein phosphatase 2A. Oncol. Rep..

[52] Hu B., Cai H., Yang S., Tu J., Huang X., Chen G. (2019). Berbamine enhances the efficacy of gefitinib by suppressing STAT3 signaling in pancreatic cancer cells. Onco. Targets Ther..

[53] Zhu X.D., Sun H.C. (2019). Emerging agents and regimens for hepatocellular carcinoma. J. Hematol. Oncol..

