# Supplementary material for: Blockade of AMPK-Mediated cAMP–PKA–CREB/ATF1 Signaling Synergizes with Aspirin to Inhibit Hepatocellular Carcinoma
Source: Cancers (Basel). 2021 Apr 6;13(7):1738. doi: 10.3390/cancers13071738 (PMC8038809; doi:10.3390/cancers13071738)

Original Western Blots

Figure 1a

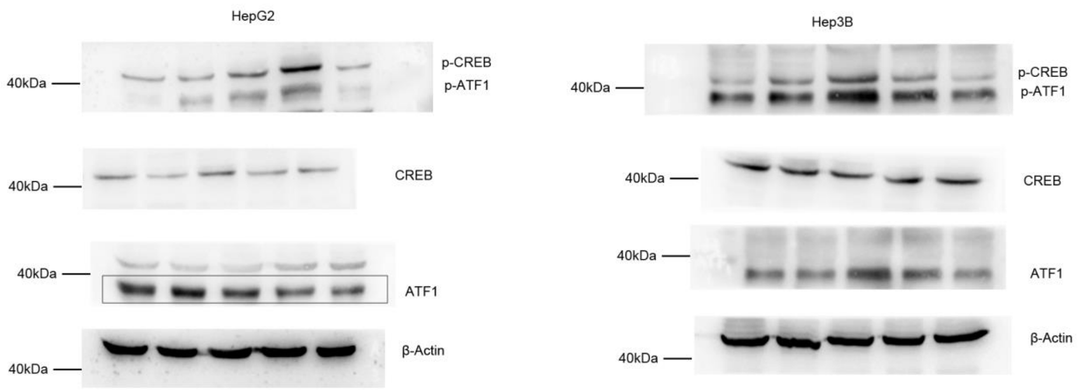

Figure 1b

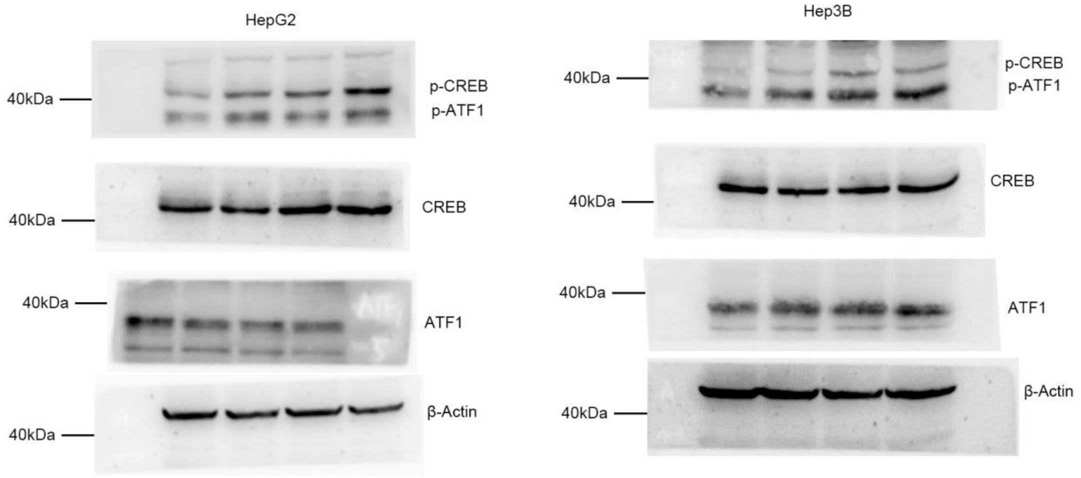

Figure 1c

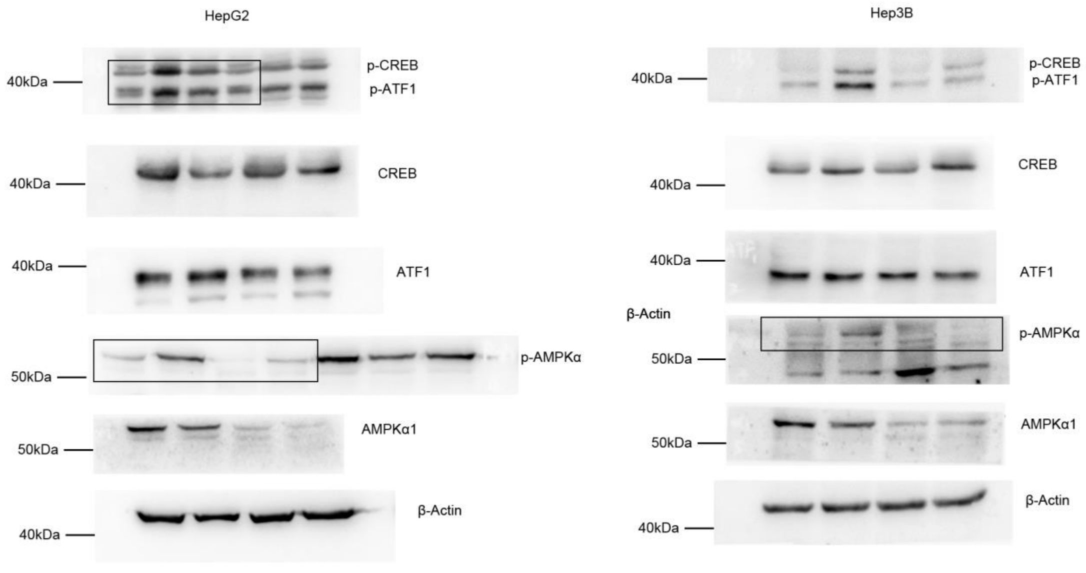

Figure 2a

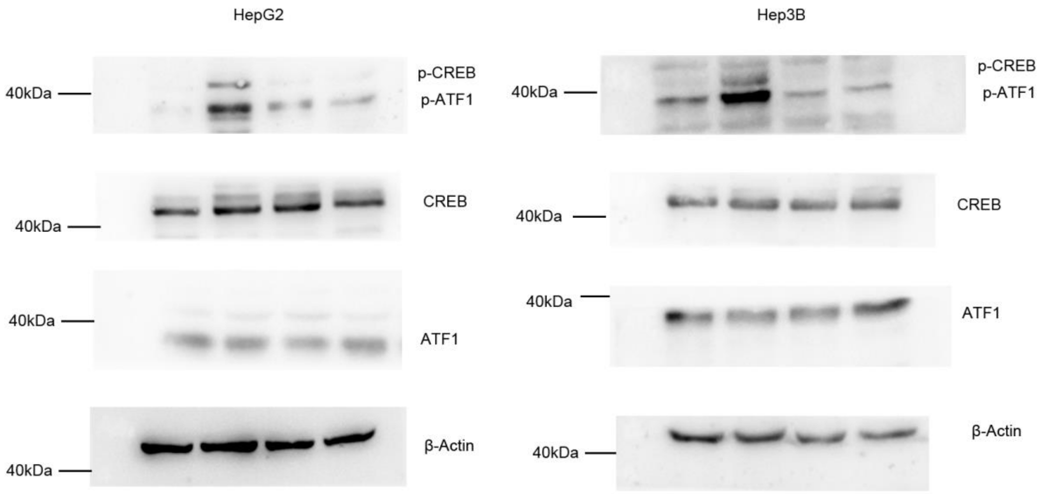

Figure 2f

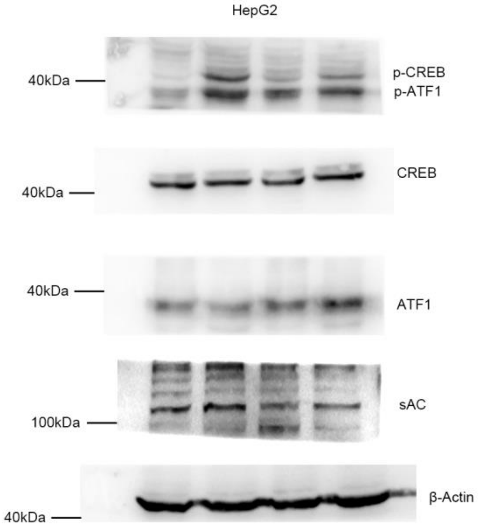

Figure 3a

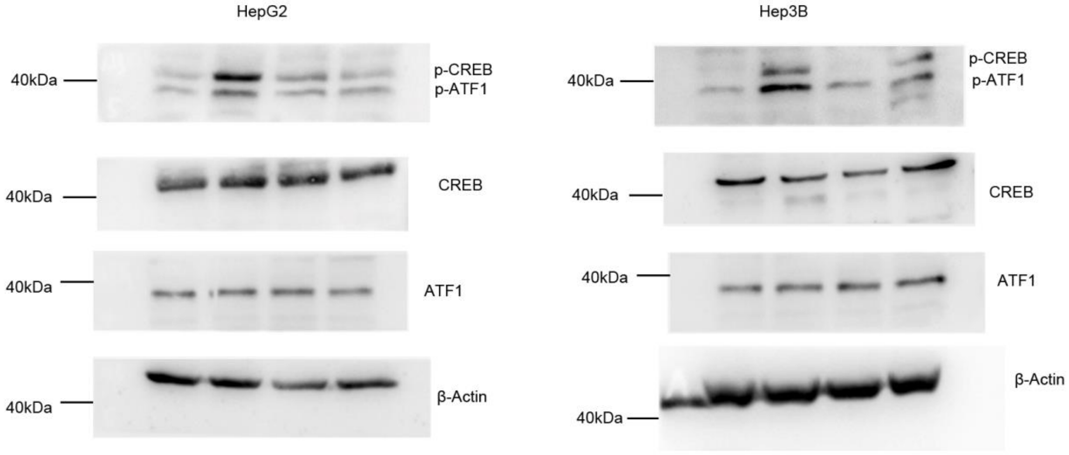

Figure 3c

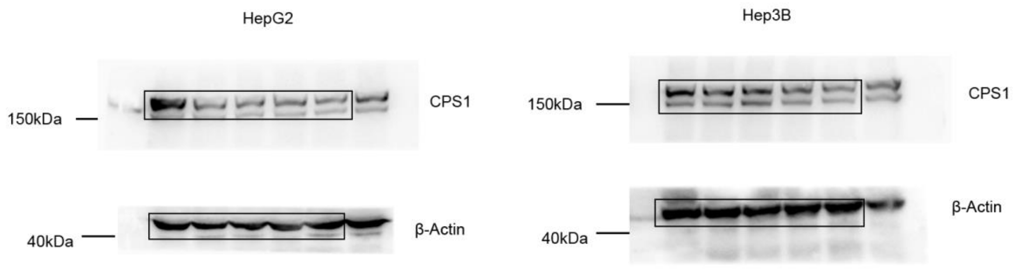

Figure 3d

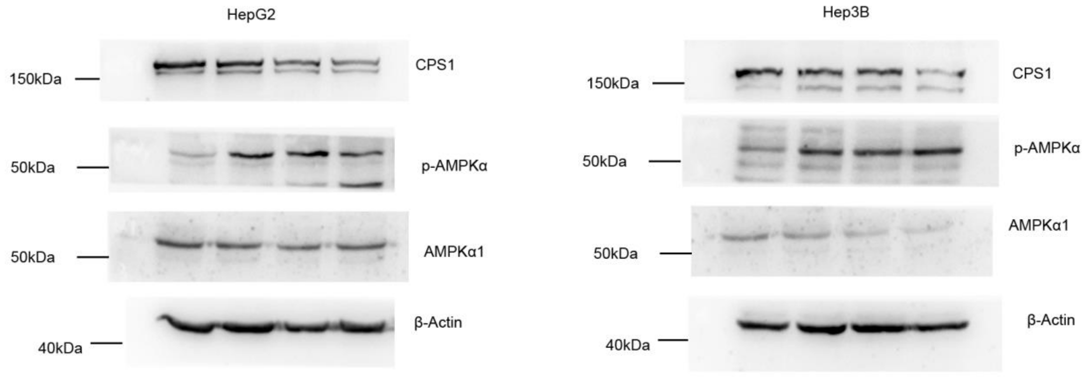

Figure 3e

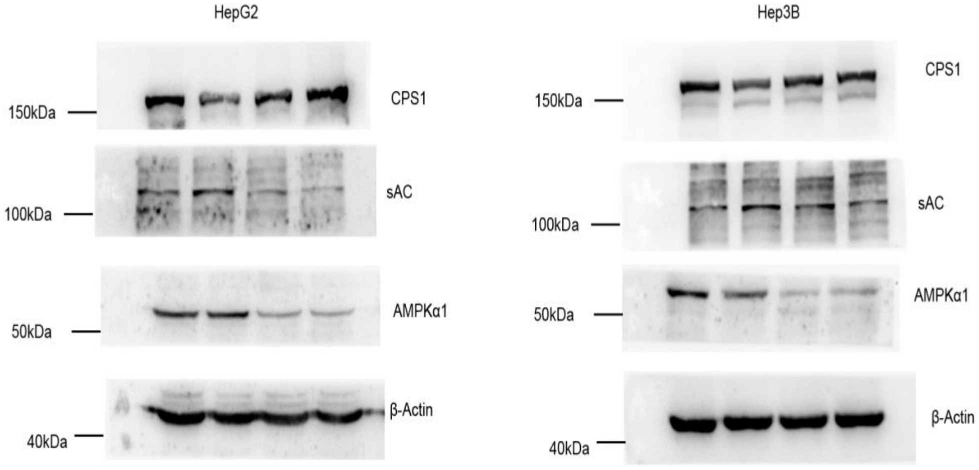

Figure 3f

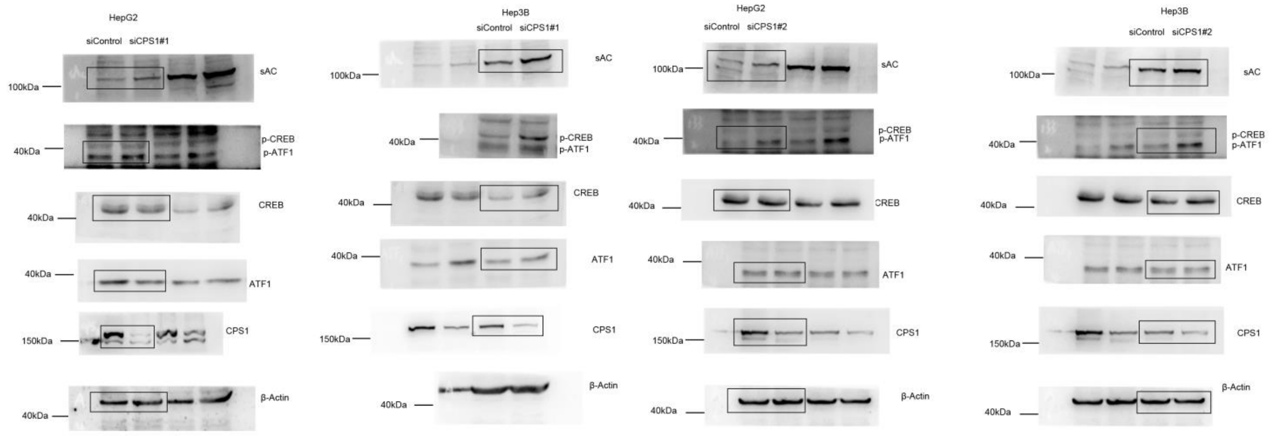

Figure 4a

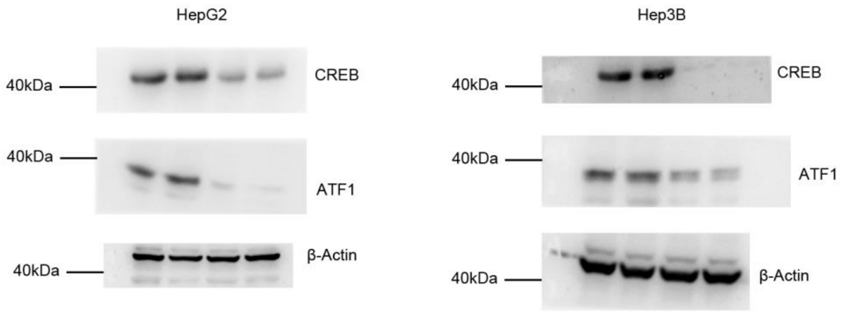

Figure 5b

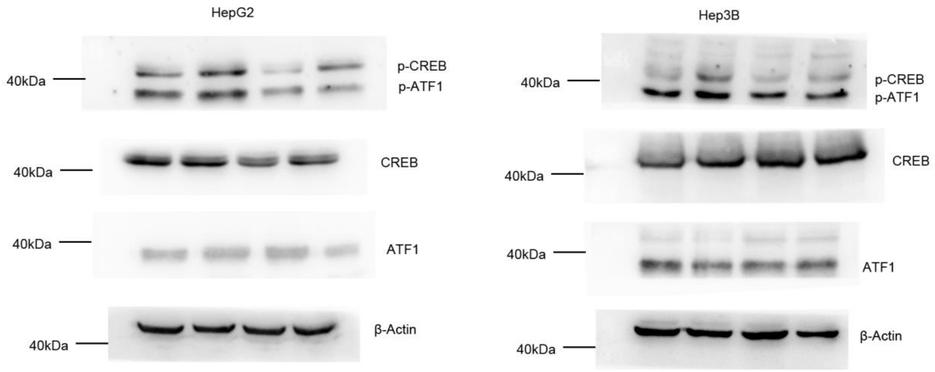

Figure 6a

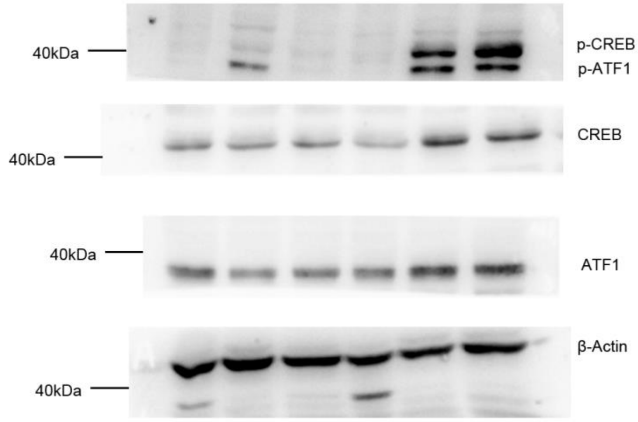

Figure 6b

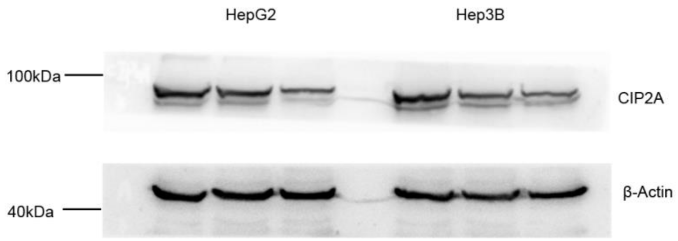

Figure 6c

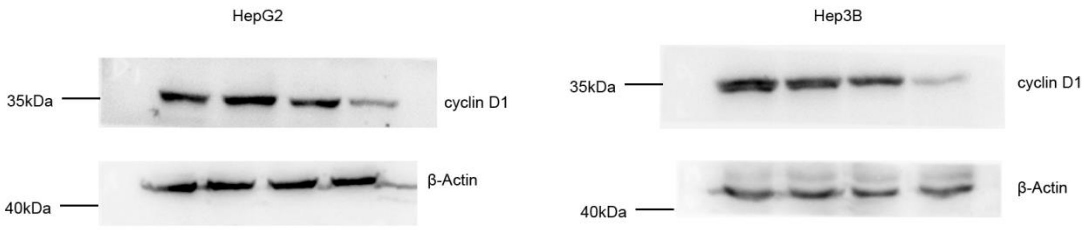

Figure 6d

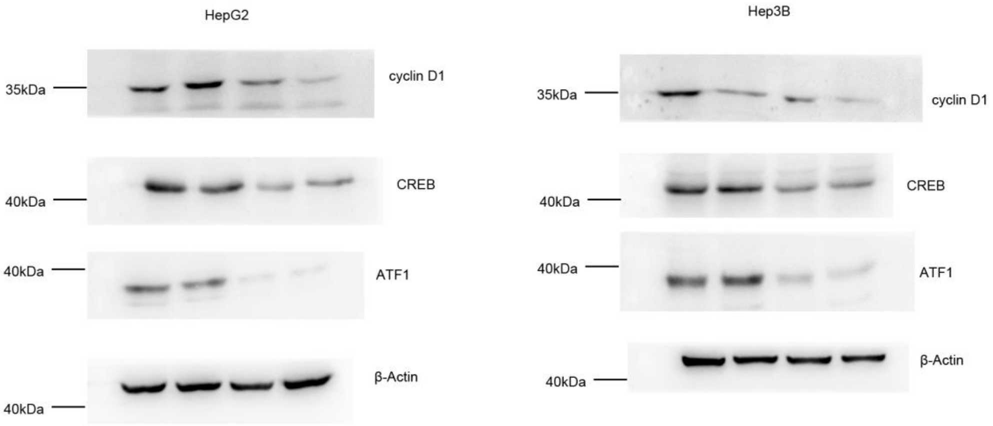

Figure S1

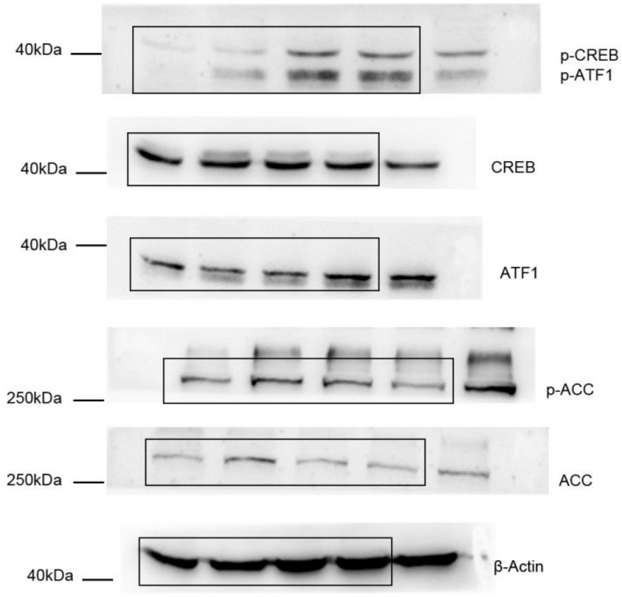

Figure S3

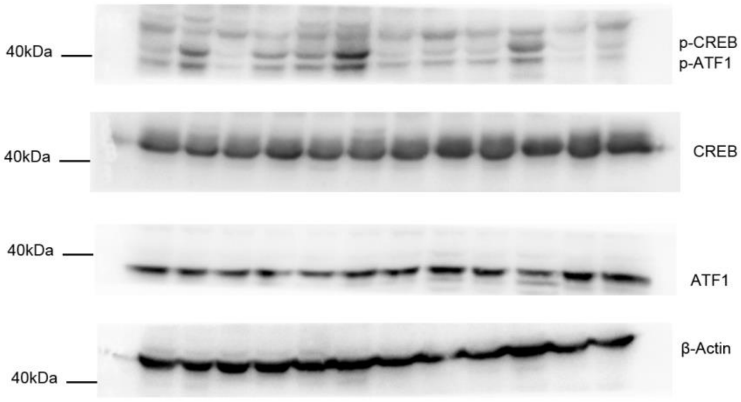

Figure S4

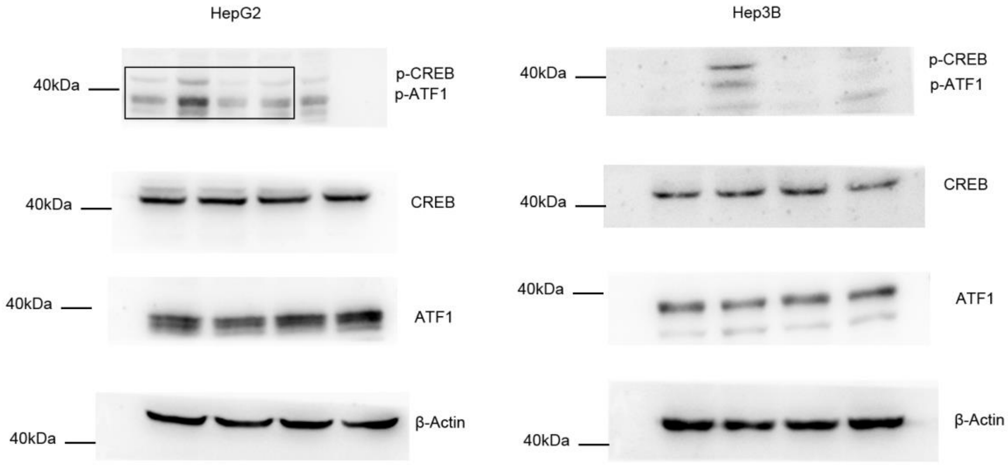

Figure S6

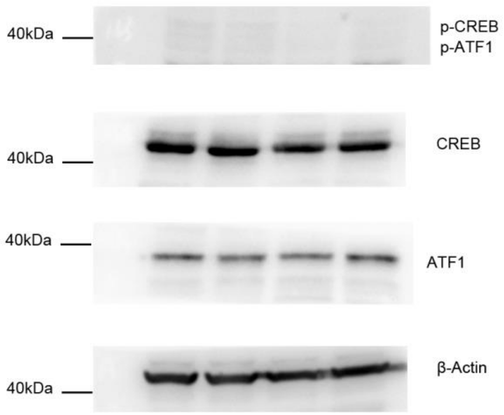

Figure S7

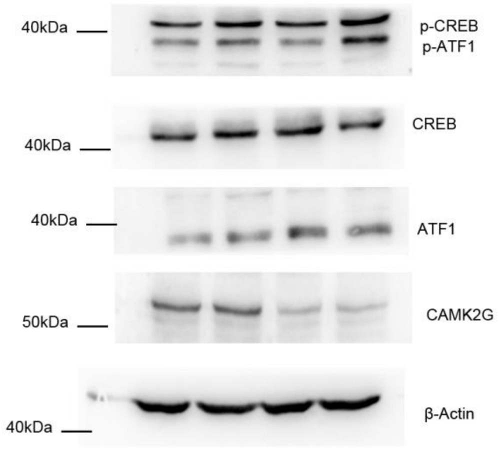

Figure S10

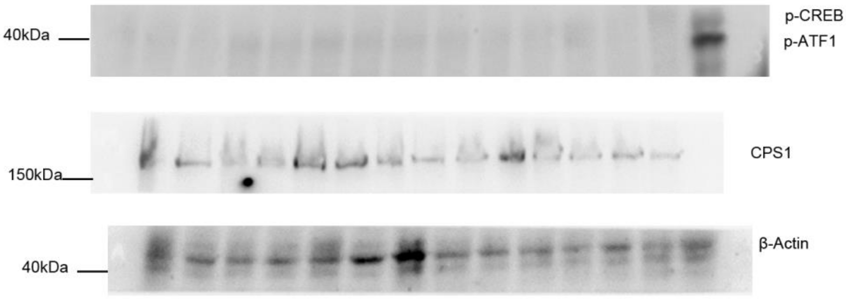

Supplement: Supplementary file 1 [file cancers-13-01738-s001.zip › 2021-04-07-suppl/File S1-Original western blots.pdf]
