# Supplementary material for: Blockade of AMPK-Mediated cAMP–PKA–CREB/ATF1 Signaling Synergizes with Aspirin to Inhibit Hepatocellular Carcinoma
Source: Cancers (Basel). 2021 Apr 6;13(7):1738. doi: 10.3390/cancers13071738 (PMC8038809; doi:10.3390/cancers13071738)
Supplement: Supplementary file 1 [file cancers-13-01738-s001.zip › 2021-04-07-suppl/Supplementary Figures.pdf]

Blockade of AMPK-Mediated cAMP–PKA–CREB/ATF1 Signaling Synergizes with Aspirin to Inhibit Hepatocellular Carcinoma

Hongying Zhang, Songpeng Yang, Jiao Wang, Yangfu Jiang

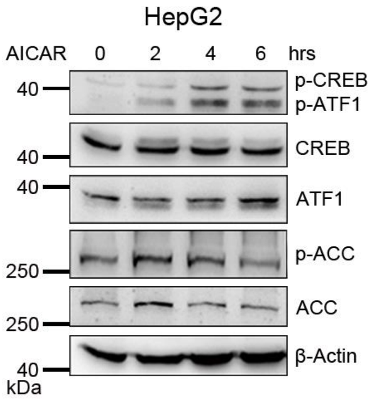

Figure S1. The AMPK agonist AICAR induces CREB/ATF1 phosphorylation in HepG2 cells. HepG2 cells were treated with 0.5 mM AICAR for indicated periods, followed by western blot analysis of indicated proteins.

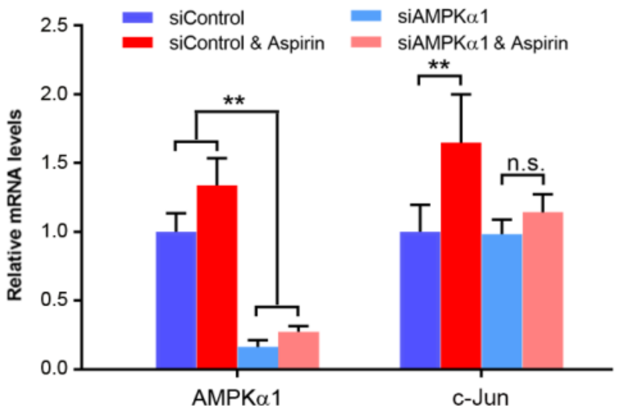

Figure S2. AMPKα1 knockdown abrogates the induction of c-Jun transcription by aspirin. Hep3B cells were transfected with siControl or siAMPKα1, and treated with or without 5 mM aspirin, followed by real-time RT-PCR analysis of indicated genes. The relative mRNA levels were plotted. Values represent mean ± SD (n = 3). **, $p < 0.01$. *n.s.*, not significant.

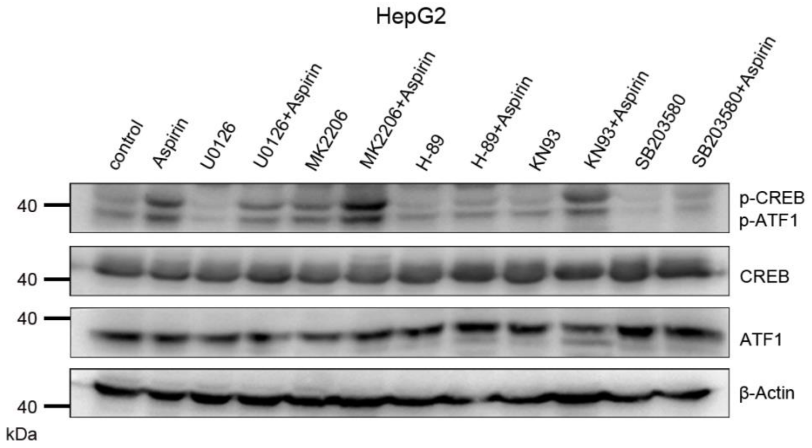

Figure S3. The effects of MEK inhibitor U0126, Akt inhibitor MK2206, PKA inhibitor H-89, CAMK inhibitor KN93 and p38 inhibitor SB203580 on the induction of CREB/ATF1 phosphorylation by aspirin in HepG2 cells. HepG2 cells were treated with 5 mM aspirin individually or in combination with indicated inhibitors for 32 hours, followed by western blot analysis of indicated proteins.

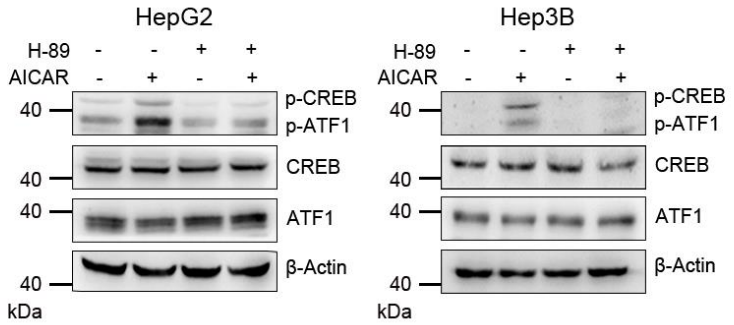

Figure S4. The effects of PKA inhibitor H-89 on the induction of CREB/ATF1 phosphorylation by AMPK agonist AICAR. HepG2 and Hep3B cells were incubated with 20 µM H-89 for 24 hours, and treated with 0.5 mM AICAR for further 6 hours, followed by western blot analysis of indicated proteins.

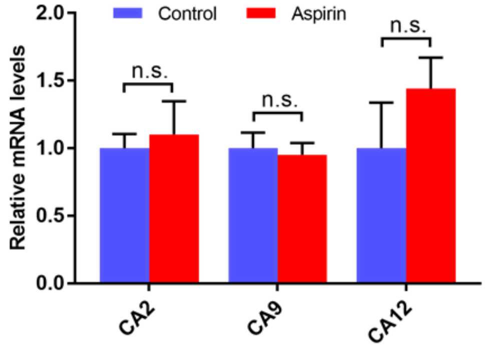

Figure S5. The effects of aspirin on CA2, CA9 and CA12 expression in HepG2 cells. HepG2 cells were treated with 5 mM aspirin for 48 hours, followed by quantitative RT-PCR analysis of CA2, CA9, and CA12 transcription. The relative mRNA levels were plotted. Values represent mean ± SD (*n* = 3). *n.s.*, not significant.

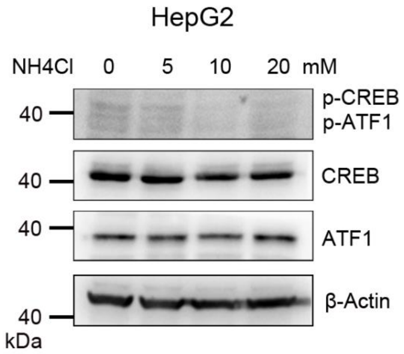

Figure S6. NH_4Cl does not induce CREB/ATF1 phosphorylation in HepG2 cells. HepG2 cells were treated with indicated concentration of NH_4Cl for 48 hours, followed by western blot analysis of indicated proteins.

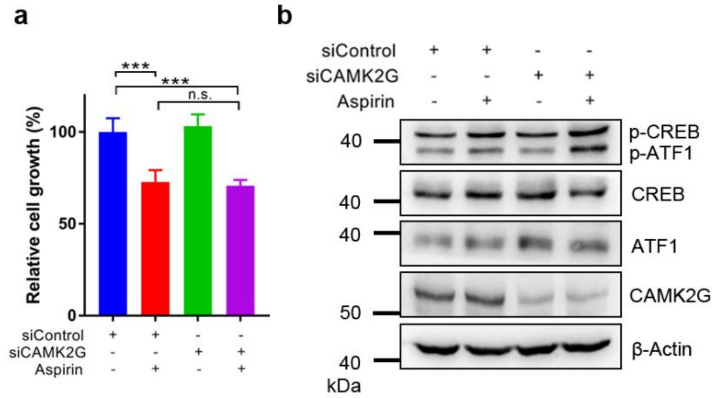

Figure S7. CAMK2G knockdown does not sensitize Hep3B cells to aspirin. **(a)** Hep3B cells were transfected with siControl or siCAMK2G, and treated with or without 5 mM aspirin for 48 hours, followed by CCK-8 assays. The relative cell growth was plotted. Values represent mean ± SD (n = 5). ***, $p < 0.001$. *n.s.*, not significant. **(b)** Hep3B cells were transfected with siControl or siCAMK2G, and treated with or without 5 mM aspirin for 48 hours, followed by western blot analysis of indicated proteins.

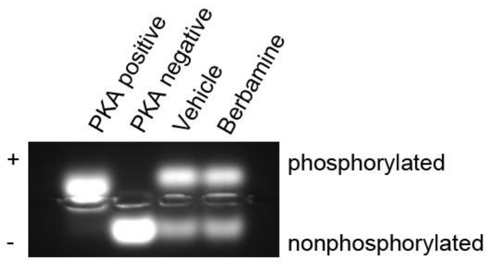

Figure S8. Berbamine does not inhibit PKA activity. HepG2 cells were treated with or without 40 μM berbamine, followed by PKA kinase assay. PKA-phosphorylated substrate migrated toward the anode (+), whereas the unphosphorylated substrate migrated toward the cathode (-).

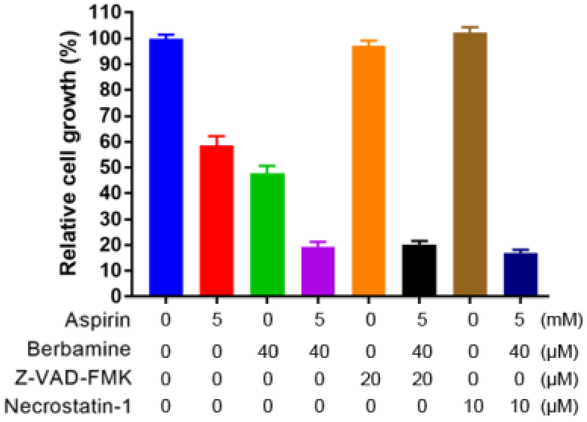

Figure S9. The caspase inhibitor Z-VAD-FMK and necroptosis inhibitor necrostatin-1 do not affect the inhibition of HepG2 cells growth by aspirin and berbamine. HepG2 cells were incubated with indicated inhibitors for 48 hours, followed by CCK-8 assays. The relative cell growth was plotted. Values represent mean ± SD (n = 5).

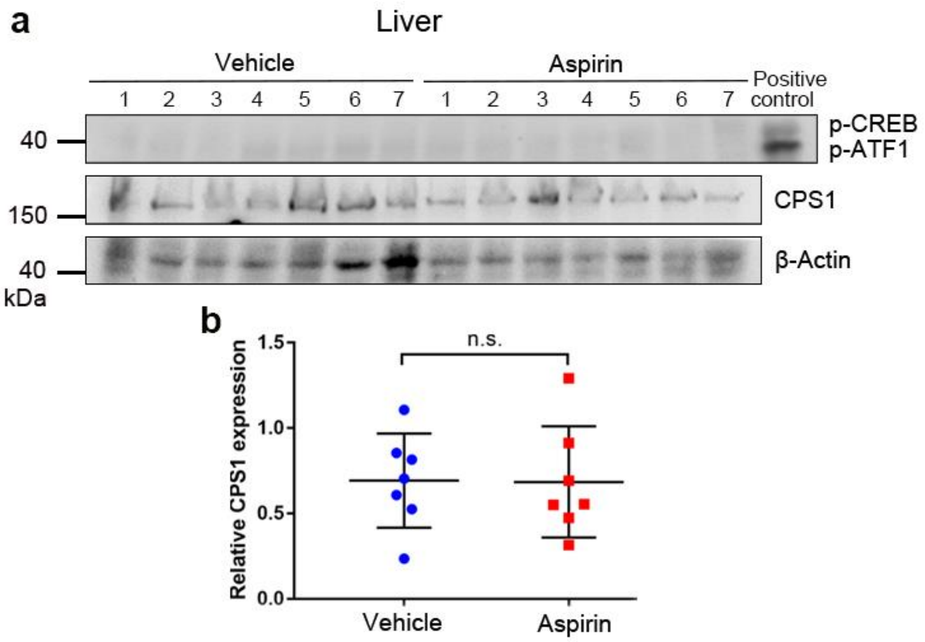

Figure S10. Aspirin does not induce CREB/ATF1 phosphorylation and inhibit CPS1 expression in mouse liver tissues. **(a)** BALB/c nude mice were treated with or without aspirin (100 mg/kg/day) for 26 days. The expression of CPS1 and the phosphorylation of CREB/ATF1 in liver tissues were detected by western blotting. **(b)** The levels of CPS1 expression were plotted. *n.s.*, not significant.

Table S1. List of the chemicals used in this study

Chemicals	Source	Cat. number
Aspirin	Sigma Aldrich	A2093
AICAR	Selleck	S1802
U0126	Selleck	S1102
MK2206	Selleck	S1078
H-89	Targetmol	T6250
KN93	Targetmol	T2606
Berbamine	Targetmol	T2920
SB203580	MedChem Express	HY-10256
Etomoxir	MedChem Express	HY-50202
Compound C	MedChem Express	HY-13418
Lactic acid	Sangon Biotech	A504045
Gefitinib	Targetmol	T1181
Sorafenib	Targetmol	T0093L
Liproxstatin-1	MedChem Express	HY-12726
Z-VAD-FMK	Targetmol	T6013
Okadaic acid	Beyotime	S1786

Table S2. List of the antibodies used in this study

Antibodies	Source	Cat. number
p-CREB(S133)/ATF1(S63)	Cell Signaling Technology	9198
p-AMPKα(T172)	Cell Signaling Technology	2535
AMPKα1	Cell Signaling Technology	2795
p-ACC(S79)	Cell Signaling Technology	11818
ACC	Cell Signaling Technology	3676
CREB	Proteintech	12208-1-AP
β-Actin	Proteintech	60008-1-AP
CPS1	Proteintech	18703-1-AP
CAMK2G	Proteintech	12666-2-AP
ATF1	Abcam	ab181569
sAC	Abcam	ab203204
cyclin D1	Abcam	ab40754
PCNA	Abcam	ab92552

Table S3. List of the siRNA sequences for RNA interference

siRNA	sequences (5'-3')
siControl	UUCUCCGAACGUGUCACGU
siCREB	GUCUCCACAAGUCCAAACA
siATF1	CGAACUACACCUUCAGCUATT
siAMPKα1	CCUCAAGCUUUUCAGGCAU
si-sAC	GAAUAUCUAGGUACAUGGA
siCPS1#1	CAAGGAATATGGTGTGAAA
siCPS1#2	GGCUUAAAGCAAGCGGAUA
siCAMK2G	CGAAAACAGGAGAUCAUUA

Table S4. List of the primer sequences for quantitative reverse-transcription polymerase chain reaction

Gene names	Primer sequences (5'-3')
GAPDH-F	ATGGGCAGCCGTTAGGAAAG
GAPDH-R	ATCACCCGGAGGAGAAATCG
c-Jun-F	ACTCGGACCTCCTCACCTCG
c-Jun-R	CTCCTGCTCATCTGTCACGTTCT
c-Fos-F	GTCTCCAGTGCCAACTTCATT
c-Fos-R	TCCTTTCCCTTCGGATTCTC
CREB-F	CTGCTCCCACCGTAACTCT
CREB-R	CTTCAATCCTTGGCACTCC
ATF1-F	TCCTCACAGAAAGCCCACG
ATF1-R	CAGCAGCAGAAACTCCAGAAT
sAC-F	GGCACTGAAGCTCCTCAACCG
sAC-R	CCTGGAGGTGGGCTCTCTTGG
CPS1-F	TGGACGCTGTTGGCAAAGATGG
CPS1-R	TGGTTTGTGTGGGCAGCATCAG
CA2-F	TCGTGGCCTCCTTCCTGAATCC
CA2-R	GCTCGCTGCTGACGCTGATG
CA9-F	GCTGCTTCTGGTGCCTGTC
CA9-R	GGGAGCCCTCTTCTTCTGATT
CA12-F	AACTCAGACCTTTATCCTGACG
CA12-R	TCCTGGCCTTTGTACTTTACAT
AMPKα1-F	ACCCATATTATTTGCGTGTA
AMPKα1-R	CTGTGGAGTAGCAGTCCCT
